# Bioinspired nano-micron hydrogel microspheres for periodontitis therapy through synergistic multi-targeted remodeling of microenvironment

**DOI:** 10.7150/thno.112782

**Published:** 2025-06-09

**Authors:** Siqi Zhou, Yuxin Zhang, Liwen Zheng, Chang Liu, Jiajun Chen, Yaxian Liu, Shidian Ran, Tong-Chuan He, Mengqin Gu, Si Wu, Fugui Zhang, Hongmei Zhang

**Affiliations:** 1 Chongqing Key Laboratory of Oral Diseases, The Affiliated Hospital of Stomatology, Chongqing Medical University, Chongqing, China.; 2 Department of Pediatric Dentistry, The Affiliated Hospital of Stomatology, Chongqing Medical University, Chongqing, China.; 3 Chongqing Municipal Key Laboratory of Oral Biomedical Engineering of Higher Education, Chongqing, China.; 4 Chongqing Municipal Health Commission Key Laboratory of Oral Biomedical Engineering, Chongqing, China.; 5 Molecular Oncology Laboratory, Department of Orthopaedic Surgery and Rehabilitation Medicine, The University of Chicago Medical Center, Chicago, Illinois, USA.

**Keywords:** periodontitis, *fusobacterium nucleatum*, berberine, bone morphogenetic protein 9, hydrogel microspheres

## Abstract

**Background:** Periodontitis is a prevalent oral inflammatory disease that leads to alveolar bone resorption and tooth loss. It is hard to control and prone to recurrence. Current treatments often fall short due to deep, tortuous periodontal pockets and antibiotic-resistant bacteria, such as *Fusobacterium nucleatum* (*F. nucleatum*). Notably, current clinical therapies fail to simultaneously fulfill three critical objectives: robust antimicrobial efficacy, potent anti-inflammatory activity, and effective periodontal regenerative capacity.

**Methods:** Inspired by the structure of the lotus flower, nano/micron-combined hydrogel microspheres (PDA/BBR@Gel@BMP9-PDLSC), encapsulating polydopamine (PDA) nanoparticles carrying berberine (PDA/BBR) and BMP9-infected PDLSCs (BMP9-PDLSC), were developed.

**Results**: Microspheres exhibited excellent biocompatibility and sustained drug release, along with significant antibacterial, anti-inflammatory, and bone tissue regenerative effects. *In vivo* studies confirmed their efficacy in treating calvaria defects and periodontitis with persistent* F. nucleatum* infection, showing superior new bone formation and anti-inflammatory effects without organ toxicity. Notably, our study demonstrated that the anti-inflammation and osteogenesis effects were due to the synergistic effects of BBR and BMP9 released by PDA/BBR@Gel@BMP9-PDLSC microspheres. RNA-Seq and Western blot analysis showed that BBR and BMP9 synergistically reduced inflammation and promoted bone formation by regulating key genes involved in TNF, TGF-β, and PPAR signaling pathways.

**Conclusions:** Our study provides a novel approach for comprehensively treating periodontitis with antibacterial, anti-inflammatory, and bone tissue regenerative effects.

## Introduction

Periodontitis is a highly prevalent oral inflammatory disease initiated by plaque microorganisms that culminates in gingival recession, alveolar bone resorption, and tooth loss 1. Besides, patients with periodontitis have an enhanced risk of systemic diseases, including diabetes, Alzheimer's disease, and rheumatoid arthritis 2. Current treatments primarily focus on preventing disease progression, such as mechanical debridement and antibiotics, which often fall short when addressing tortuous deep periodontal pockets and antibiotic-resistant bacteria 3-6. *Fusobacterium nucleatum* (*F*. *nucleatum*) is a dominant antibiotic-resistant bacterium that has pivotal roles in dental plaque biofilm formation and progression of periodontitis 7. The presence of *F*. *nucleatum* means that periodontitis is harder to control and more prone to recurrence 8. Minocycline hydrochloride is a commonly used antibacterial agent to combat biofilm in deep periodontal pockets, but it is sometimes limited by bacterial resistance and side effects 9. Therefore, natural-source drugs or other materials are needed as an alternative to antibiotics to combat or eradicate *F*. *nucleatum* and effectively improve the poor microenvironment.

Traditional Chinese medicine (TCM) has a long history in periodontitis treatment, with its safety and minimal side effects 10-12. Of the many TCM components, berberine (BBR) has garnered considerable attention due to its significant antimicrobial and anti-inflammatory effects 13-15. As a natural antimicrobial, BBR has substantial advantages over synthetic antibiotics with a lower likelihood of developing resistance and significant gut-protective effects 15-17. BBR suppresses biofilm formation and virulence factor expression by restraining bacterial quorum sensing and disrupting cell membranes, allowing bacteriostatic function at low concentrations and bactericidal function at high concentrations 16. Moreover, BBR modulates multiple anti-inflammatory pathways and enhances stem cell proliferation and differentiation 15, 18-20. Therefore, BBR is a multidimensional TCM component that effectively combats bacterial infection and regulates anti-inflammatory responses 21-24. However, its poor water solubility and low bioavailability restrict applications against *F. nucleatum* infection in periodontitis 25. Polydopamine (PDA) nanoparticles with robust permeability and retention roles can facilitate drug loading via physical absorption and may act as carriers to enable targeted delivery of BBR and significantly enhance drug utilization 26.

Current clinical strategies primarily focus on antimicrobial therapy and inflammatory control, but cannot realize the regeneration of periodontal tissues. In general, the process of periodontal regeneration is highly organized and relies on the complex interaction of stem cells, biofactors, and signaling pathways. It is well known that bone morphogenetic protein 9 (BMP9) is a secretory protein with crucial roles in regulating osteogenesis and anti-inflammatory processes 27-30. Studies have demonstrated that periodontal ligament stem cells (PDLSCs) infected with BMP9 exhibited superior bone regeneration capabilities compared to other types of dental mesenchymal stem cells and activated critical intracellular signaling molecules, such as Smads, to regulate osteogenic gene expression, promote osteoblast differentiation, and inhibit osteoclast activity 31-33. Hence, BMP9-infected PDLSCs are potentially valuable in clinical applications, such as bone defect repair, suggesting their use in regenerative treatments for periodontitis-induced bone tissue defects. Thus, BMP9-infected PDLSCs, in combination with PDA nanoparticles carrying BBR, may complement each other and more effectively modulate the local periodontitis microenvironment.

Apart from appropriate “drug” selection, effective “drug” delivery to complex and irregular lesions is also crucial. Current oral treatment administration routes have many limitations, such as controlling drug concentrations, saliva interference, poor patient experiences, and inefficient delivery to irregular lesion areas 34-36. Hence, a more precise and stable personalized drug delivery system is required. Integrating BMP9-infected PDLSCs and PDA nanoparticles carrying BBR into advanced biomaterials for delivery to periodontal lesions presents an innovative and potentially effective solution. The evolution of natural living organisms has provided specific architectures to achieve high environmental adaptability, thus offering valuable inspiration for building high-performance materials 37. The lotus, named the “dual-life flower” by the Chinese, embodies a symbiotic relationship between the lotus seeds and flowers. This biological symbiosis inspired our design of hydrogel microspheres, which can realize the unique interplay between PDA nanoparticles carrying BBR and BMP9-infected PDLSCs. More importantly, the lotus seedpod, which hides in petals and contains rich seeds, realizes the symbiosis of lotus flowers and seeds. Thus, lotus seedpod-inspired material gelatin methacrylate (GelMA) hydrogel microspheres may act as a significant intermediary to integrate BMP9-infected PDLSCs and PDA nanoparticles carrying BBR.

**Scheme 1:**
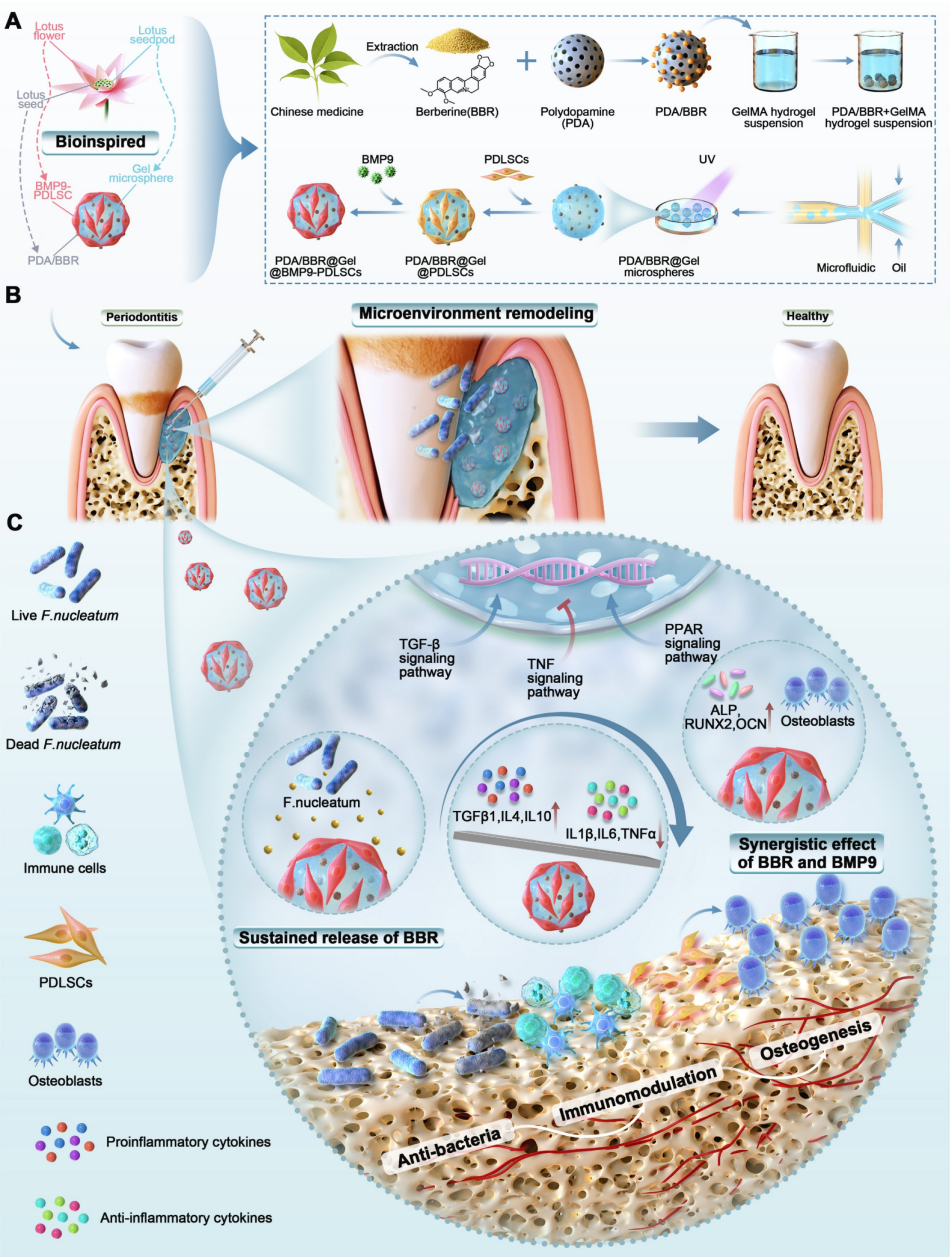
Schematic illustration of PDA/BBR@Gel@BMP9-PDLSC microsphere formation and its multifunctional effects in treating periodontitis. (A) Fabrication process of PDA/BBR@Gel@BMP9-PDLSC microspheres inspired by the lotus. (B) *In situ* injection of PDA/BBR@Gel@BMP9-PDLSC microspheres for periodontitis treatment. (C) Multifunctional effects (anti-bacteria, immunomodulation, and osteogenesis) and mechanisms of PDA/BBR@Gel@BMP9-PDLSC microspheres for remodeling periodontal microenvironment.

Based on these premises, we designed a safer and multifunctional material adapted to complex periodontitis lesions and mixed PDA nanoparticles with BBR. Then, we combined them with GelMA hydrogel using microfluidics to create injectable microspheres. BMP9-infected PDLSCs were next introduced onto the surface of hydrogel microspheres. Thus, nano/micron-combined hydrogel microspheres were developed to co-deliver BBR and BMP9. The study design is illustrated in Scheme 1. These microspheres provided excellent biocompatibility and sustained release, effectively targeting *F*. *nucleatum*, promoting osteogenesis, reducing inflammation *in vitro* and *in vivo*, and eventually facilitating microenvironment remodeling in periodontitis. RNA sequencing (RNA-seq) revealed potential mechanisms by which BBR and BMP9 synergistically regulated key genes involved in osteogenesis and anti-inflammation. This regulation was achieved by activating the TGF-β and PPAR signaling pathways and inhibiting the TNF signaling pathway. Taken together, our research presents a novel approach for comprehensively treating periodontitis by combining BBR and BMP9 in a bioinspired delivery system, renewing the two “traditional drugs” for improving disease prognoses.

## Materials and methods

### Polydopamine (PDA)/BBR nanoparticle preparation

PDA was synthesized as previously reported 38. First, 0.36 g each of Pluronic F127 (Sigma-Aldrich, USA) and TMB (MedChemExpress, USA) were dissolved in 100 mL of 50% (v/v) ethanol aqueous solution under magnetic stirring for 30 min. Then, 0.1 g of Tris (Sigma-Aldrich, USA) was dissolved in 10 mL distilled water, followed by the addition of 60 mg dopamine monomer (DA; MedChemExpress) under continuous stirring in the dark at room temperature for 24 h. The nanoparticles were harvested by centrifugation at 4,000 rpm for 5 min and sequentially washed with anhydrous ethanol and acetone. For BBR (MedChemExpress, USA) loading, the compound was introduced into the PDA-ethanol solution followed by 10 min of vortex mixing at 2,000 rpm. After 72 h of static incubation at room temperature, PDA/BBR nanoparticles were collected by centrifugation at 11,000 rpm for 10 min.

### GelMA (Gel) microspheres loaded with PDA/BBR

The lyophilized gelatin methacryloyl (GelMA, Aladdin, China) was reconstituted in a 4% (w/v) lithium phenyl-2,4,6-trimethylbenzoylphosphinate (LAP, MedChemExpress, USA) aqueous solution at 100 mg/mL, incubated at 37°C for 30 min to ensure complete hydration. Subsequently, the PDA/BBR suspension was incorporated into the GelMA precursor through vortex mixing. To prepare uniformly sized PDA/BBR@Gel microspheres, microfluidics was used according to a previous method 39. Briefly, the microfluidic device was assembled using two separate syringe pumps (LongerPump, China) and a coaxial electrospinning nozzle, which featured an inner needle of 25 G and an outer needle of 18 G. The oil phase was mineral oil (Rhawn, China) containing 10% (v/v) Span 80 (Rhawn, China). The mixed solution of GelMA and PDA/BBR was used as the water phase. The water phase was introduced into the inner needle, while the oil phase was delivered into the outer needle via the syringes. The water phase flow rate was maintained at a constant speed of 20 μL/min, and the oil phase flow rate was adjusted to 700 μL/min to facilitate the preparation of microspheres.

### Material characterization

Scanning electron microscopy (SEM, Hitachi, Japan) was applied to capture surface structure and pore morphology. Fourier-transform infrared spectroscopy (FTIR, Waltham, USA) was used to confirm successful BBR loading into PDA nanoparticles by comparing characteristic peaks of PDA, BBR, and PDA/BBR. Particle size distribution and material differences were analyzed using Image J software. The zeta potential of various nanoparticles was assessed with Zetasizer Pro system (Malvern Panalytical, UK). The swelling ratio of lyophilized PDA/BBR@Gel microspheres was determined by measuring their mass after immersion in phosphate buffered saline (PBS). To test the mechanical performance of hydrogel microspheres, a universal testing machine (MTS Systems, China) was employed to measure the mechanical characteristics of the hydrogel. The compression rate was maintained at a constant 1 mm/min, maximum strain = 90 %. The compression module was derived based on the slope of the linear region of stress-strain result. The rheological characterizations of the hydrogels were tested by a rotational rheometer (Anton Paar, China) with frequency sweep at 25 °C. The storage modulus (G') and loss modulus (G") of the hydrogels were evaluated by dynamic frequency sweep test at 0.1-100 rad/s.

### Drug release and degradation behavior of PDA/BBR@Gel microspheres *in vitro*

To assess the sustained release effects of PDA/BBR@Gel microspheres, they were incubated in PBS (pH 6.3 and 7.4, both with 0.1 U/mL collagenase II) and placed in a shaker (37 °C, 80 rpm) to simulate physiological and inflammatory oral conditions, respectively. The supernatant was then collected at predetermined timepoints to measure BBR concentrations on a Multimode Plate Reader (PerkinElmer, EnSpire, USA).

To investigate the degradation behavior of the microspheres, PDA/BBR@Gel microspheres were also placed in 2 mL of PBS (pH 6.3 and 7.4, both with 0.1 U/mL collagenase II) and placed in a shaker (37 °C, 80 rpm) to simulate physiological and inflammatory oral conditions 40-42. On the predetermined days, the degradation solution was removed and the remaining microspheres were weighed (W_t_). The remaining weight (%) of the microspheres was computed using the following formula: Weight of remaining microspheres (%) = W_t_ / W_0_ × 100%, where W_0_ is the initial weight of the fully swollen microspheres.

### Adhesion performance of PDA/BBR@Gel microspheres

Rat maxillary gingival tissues were isolated to determine the adhesion property of PDA/BBR@Gel microspheres to biological tissues. After attaching Gel microspheres (dyed in red ink) and PDA/BBR@Gel microspheres (dyed in black ink) in the isolated rat maxillary gingival tissue, a specific flow rate of water was used for some time. The remaining adhesion area of microspheres was observed and captured.

### Isolation and culture of periodontal ligament stem cell (PDLSC)

Dental stem cell isolation and culture methods were previously described 33, 43. Briefly, PDLSCs were obtained from discarded human premolar teeth, extracted during orthodontic treatment, and with patient consent. Using teeth for scientific purposes was approved by the Ethics Committee of The Affiliated Hospital of Stomatology, Chongqing Medical University [CQHS-REC-2024 (LSNo.31)]. First, periodontal ligament tissues were digested in Type I collagenase (3 mg/mL, Sigma-Aldrich) and cultured in α-Modified Eagle Medium (MEM) (Hyclone, USA) supplemented with 10% fetal bovine serum (FBS, AuGenex, Australia) and 1% penicillin/streptomycin at 37 °C in 5% CO_2_ to generate primary PDLSCs. Lipopolysaccharide (LPS, 10 μg/mL, Beyotime, China) was added to the medium to simulate an inflammatory environment.

### The construction and amplification of BMP9 and GFP recombinant adenoviruses

The 293pTP cell line was used for adenovirus construction and amplification 44. 293pTP cells were cultured in Dulbecco's Modified Eagle Medium (DMEM) (Hyclone, USA) plus 10% FBS (AuGenex, Australia) and 1% penicillin/streptomycin at 37 °C in 5% CO_2_. Recombinant adenoviruses expressing BMP9 (Ad-BMP9) and green fluorescent protein (Ad-GFP) were generated using the AdEasy Technology, as previously described 45-47.

### The construction of PDA/BBR@Gel@BMP9-PDLSC microspheres

First, 20 mg of PDA/BBR@Gel microspheres and PDLSCs (2 × 10⁴ cells/well) were seeded into 24-well plates. Following a 2-hour adhesion period, cells were transduced with Ad-BMP9 or Ad-GFP at a multiplicity of infection (MOI) of 50 (achieving 80% infection efficiency) along with polybrene (10 μg/mL, Solarbio, China) to enhance viral transduction efficiency. After 8 h, the medium was changed for continuous culturing, and infected cells were examined under fluorescence microscopy (Nikon, Japan) at 24 h post stimulation.

### Live/dead staining

After 5- and 7-days co-culturing PDLSCs with PDA/BBR@Gel microspheres loaded with different concentration of BBR (0 µg/mL, 1 µg/mL, 2 µg/mL, and 3 µg/mL), live/dead cell staining (Beyotime, China) was performed to detect PDLSC viability. After washing in PBS, cells were stained in Calcein-AM/PI solution for 30 min according to manufacturer's instructions. Live/dead cell staining corresponding to green/red fluorescence, respectively, was observed under fluorescence microscopy, with positively stained green/red cell quantification performed using Image J software.

### Cell counting kit-8 (CCK-8) assay

After co-culturing PDLSCs with PDA/BBR@Gel microspheres, cell proliferation was analyzed using a CCK-8 kit (Beyotime, China) on days 1, 3, and 5. Cells were treated with a CCK-8 stock solution diluted in α-MEM (10:1 ratio) and incubated at 37 °C for 2 h. Absorbance was measured at 450 nm on a Multimode Plate Reader (PerkinElmer, EnSpire, USA).

### Cell spread and adhesion

After 3-, 5-, and 7-days co-culturing PDLSCs with PDA/BBR@Gel microspheres, cell cytoskeletons and nuclei were stained with Actin-Tracker Red-555 fluorescent dye and 4',6-diamidino-2-phenylindole (DAPI) (Beyotime, China), respectively. Cell adhesion and spread on PDA/BBR@Gel microsphere surfaces was examined under fluorescence microscopy (Nikon, Japan).

### Alkaline phosphatase (ALP) detection

PDLSCs were cultured with different microsphere groups for 7 days. ALP staining and quantitative analyses were performed using 5-Bromo-4-chloro-3-indolyl phosphate (BCIP) / Nitrotetrazolium Blue chloride (NBT) ALP color development (Beyotime, China) and ALP assay kits (Nanjing Jiancheng, China). To determine the most suitable BBR concentration for osteogenic differentiation, four microsphere groups loaded with different concentration of BBR (0 µg/mL, 1 µg/mL, 2 µg/mL, and 3 µg/mL) were established. Next, the osteogenic and anti-inflammatory properties of the microspheres were examined through ALP expression analysis within a lipopolysaccharide (LPS)-induced inflammatory microenvironment (10 µg/mL LPS, 24 h). Experimental groups were as follows: PDLSCs (Blank), PDA/BBR@Gel@PDLSCs, PDA/BBR@Gel@GFP-PDLSCs, and PDA/BBR@Gel@BMP9-PDLSCs.

### Calcium nodule and mineral salt staining

On day 21 after co-culturing PDLSCs with different microsphere groups, calcium nodules and mineral salts formed by cells were stained using Alizarin Red staining (ARS) solution (Solarbio, China). Stained calcium deposits were documented under bright field microscopy (Leica, Germany). After the staining had dried, stained calcium nodules were dissolved in cetylpyridinium chloride and absorbances quantitatively recorded using a Multimode Plate Reader. Experimental groups were similarly designated as for ALP staining section.

### *F. nucleatum*, *Porphyromonas gingivalis* (*P. gingivalis*) and *Aggregatibacter actinomycetemcomitans* (*A. actinomycetemcomitans*) resuscitation and culture

A lyophilized *F*. *nucleatum* strain (ATCC25586, Fenghui Bio, China), *P. gingivalis* strain (ATCC33277, Fenghui Bio, China) and *A. actinomycetemcomitans* strain (ATCC29523, Biofeng, China) was aseptically dissolved in PBS and inoculated onto Columbia blood agar plates (Shiyigou, China), respectively. After cultured in a 37 °C anaerobic incubator, a single colony was inoculated into Brain-Heart Infusion Broth (BHI) liquid medium (Solarbio, China) and continuously in a 37 °C anaerobic incubator for amplification.

### *F. nucleatum* Gram staining

After a single colony of the revived *F. nucleatum* had grown, a number of colonies was picked and dissolved in 100 μL of saline. The mixed bacterial suspension was then carried out for Gram staining according to the instructions of manufacturer (Solarbio, China). Stained slides were recorded under bright field microscopy (Leica, Germany).

### Bacterial growth curves

To generate bacterial growth curves, 200 mg of PDA@Gel or PDA/BBR@Gel microspheres were added to a 40 mL *F*. *nucleatum* suspension (10^8^ colony forming units (CFU)/mL) and co-cultured for 72 h. Then, 1 mL of the suspension was collected at predetermined timepoints to measure absorbance values on a Multimode Plate Reader. Growth curves were plotted for all experimental groups.

### Biofilm staining

We added 10 mg of PDA@Gel or PDA/BBR@Gel microspheres to a 200 μL of *F*. *nucleatum* suspension (10^8^ CFU/mL), *P. gingivalis* suspension (10^8^ CFU/mL) or *A. actinomycetemcomitans* suspension (10^8^ CFU/mL) and co-cultured them for 3 days (*F. nucleatum* and *A. actinomycetemcomitans*) or 5 days (*P. gingivalis*), after which biofilms were stained in 0.1% crystal violet (Solarbio, China). After the stain had dried, biofilms were dissolved in glacial acetic acid and absorbances quantitatively analyzed on a Multimode Plate Reader.

### Analyzing antibacterial effects

We added 10 mg of PDA@Gel or PDA/BBR@Gel microspheres to a 200 μL of *F*. *nucleatum* suspension (10^8^ CFU/mL) and co-cultured them for 3 days. Bacteria were stained using the SYTO9/PI staining kit (Bestbio, China) and imaged under laser confocal microscopy (Leica, Germany) to compare antibacterial effects between groups.

### RNA isolation, reverse transcription, and real-time quantitative PCR (RT-qPCR)

Total RNA was extracted from different PDLSCs and mice periodontal tissue-treatment samples using TRIzol (Thermo Fisher Scientific, USA). Total RNA (500 ng) was reverse-transcribed into cDNA using the 5x PrimeScript RT Master Mix (Takara, Japan). RT-qPCR was performed in a 10 μL reaction mixture composed of cDNA, TB Green (Takara, Japan), and primers. Gene expression was determined using a RT-qPCR detection system (BIO-RAD, USA). Primer sequences are shown in Table S1 and S2.

### RNA-Seq

RNA was isolated from PDLSCs co-cultured with different microsphere groups (for 5 days) using TRIzol (Thermo Fisher Scientific, USA). The RNA library construction and sequencing were conducted by Shanghai Majorbio Bio-Pharm Biotechnology Co., Ltd. After quantification using a Qubit 4.0, library sequencing was performed on a NovaSeq X Plus platform (PE150) using a NovaSeq reagent kit. Differential gene expression (DEG) analysis was performed using DESeq2. DEGs were identified based on *p*-values < 0.05 and absolute log2-fold changes ≥ 1. Gene Ontology (GO) enrichment and Kyoto Encyclopedia of Genes and Genomes (KEGG) pathway analyses on DEGs were performed using Goatools and Python scipy software, respectively.

### Western blotting analysis

PDLSCs were seeded and treated as in the RNA-Seq experiment. Total protein from cells were extracted in RIPA lysis buffer (Beyotime, China) containing 1% Protease and Phosphatase Inhibitor Cocktail (Beyotime, China). Equal amounts of protein were detached by a 10% SDS-PAGE gel and then transferred onto a 0.45 µm PVDF membrane (Millipore, USA). The membrane was furthermore blocked with 5% BSA in TBST for 2 h and subsequently incubated with antibodies. The visualization of immunological assays was carried out using the Enhanced Chemiluminescence (ECL) Detection Reagent (Beyotime, China). Primary antibodies were used as follows: anti-PPARγ antibody (Proteintech, China), anti-NF-κB antibody (CST, USA), anti-phosphorylated NF-κB antibody (CST, USA), anti-SMAD1 antibody (CST, USA), anti- phosphorylated SMAD1/5 antibody (CST, USA) and anti-GAPDH antibody (Proteintech, China).

### Animal studies

8-week-old male Sprague-Dawley (SD) rats and male C57BL/6J mice were purchased from Beijing Vital River Laboratory Animal Technology Co., Ltd. All animal studies complied with local animal care guidelines and the approval of all procedures was granted by the Ethics Committee of The Affiliated Hospital of Stomatology, Chongqing Medical University [CQHS-REC-2024 (LSNo.31)].

Cranial defect models were established in 8-week-old male SD rats. Animals were randomly divided into PDA@Gel@PDLSC, PDA@Gel@GFP-PDLSC, PDA@Gel@BMP9-PDLSC, PDA/BBR@Gel@PDLSC, PDA/BBR@Gel@GFP-PDLSC, and PDA/BBR@Gel@BMP9-PDLSC microsphere groups (five rats/group). Animals were anesthetized via isoflurane inhalation. After shaving cranial hair and cleaning the area with 10% povidone-iodine, an incision was made in the cranial skin. A trephine drill was used to create holes between the anterior and posterior fontanelles on both sides of the sagittal suture, while cooling with saline to avoid damaging the dura mater. After a 5 mm diameter circular cranial bone defect was constructed, test materials were injected into the defect. The incision was closed using sterile absorbable sutures, and erythromycin ointment applied to the wound surface to prevent postoperative infection. After 4 weeks, animals were sacrificed with CO_2_ Euthanasia System (30-70% rate of the CO_2_ replacement per minute) and skulls were collected and fixed in 4% paraformaldehyde.

A periodontitis mouse model was established using 8-week-old male C57BL/6J mice, randomly divided into health, ligation without treatment, ligation with PDA@Gel@PDLSC, ligation with PDA@Gel@GFP-PDLSC, ligation with PDA@Gel@BMP9-PDLSC, ligation with PDA/BBR@Gel@PDLSC, ligation with PDA/BBR@Gel@GFP-PDLSC and ligation with PDA/BBR@Gel@BMP9-PDLSC microsphere groups (five mice/group). Animals were anesthetized with an intraperitoneal injection of 0.3% sodium pentobarbital (Sigma-Aldrich, USA). Next, bilateral maxillary second molars were ligated using minimally invasive forceps and sterile silk and injected with *F.nucleatum* suspension. Microsphere materials were injected daily into periodontal areas in these molars. After 7 days of continuous *F.nucleatum* and microsphere injections, animals were sacrificed with CO_2_ Euthanasia System (30-70% rate of the CO_2_ replacement per minute). Maxillary alveolar bones and gingival samples were collected and fixed in 4% paraformaldehyde. For RNA extraction, gingiva was immediately frozen in liquid nitrogen.

### Micro-CT analysis

Skull samples and maxillary alveolar bone samples were scanned using a micro-CT system (Scanco Medical, Switzerland) at 70 kVp and 114 uA with a 15 μm voxel size. Three-dimensional (3D) reconstructions were performed using Mimics Research 19.0 software (Materialise, Belgium) and quantitative analyses were conducted based on micro-CT system packaged software. A region of interest (ROI) of the bone defect was selected. The bone volume/ total volume (BV/TV), trabecular number (Tb.N), trabecular thickness (Tb.Th) and trabecular separation (Tb.Sp) of the ROI were analyzed using the software.

### Histology and immunofluorescence analyses of animal studies

Skull samples and maxillary alveolar bone samples after decalcification in 10% Ethylene Diamine Tetra acetic Acid (EDTA) as well as heart, liver, spleen, lung, and kidney tissues were embedded in paraffin blocks and sectioned into 5 μm slices. To assess *in vivo* microsphere biocompatibility, heart, liver, spleen, lung, and kidney tissues were collected from the PDA/BBR@Gel@BMP9-PDLSC microsphere group and stained with hematoxylin & eosin (H&E) (Solarbio, China). To demonstrate the tissue repair ability, skull sample sections were stained with H&E and Masson's trichrome staining kits (Solarbio, China) according to manufacturer's instructions. Then, in order to assess the histological changes and osteoclastic activity, maxillary alveolar bone sample sections were stained with H&E and tartrate-resistant acid phosphatase (TRAP) (Solarbio, China). The number of osteoclasts in the alveolar bone was further quantified. All sections were observed and recorded under bright field microscopy.

Maxillary alveolar bone sample sections were further assessed with immunofluorescence staining as previously described 48. Rabbit monoclonal anti-OCN antibody (1:200, Abclonal, China) and mouse monoclonal anti-IL-1β antibody (1:200, Santa Cruz, USA) were used as primary antibodies. Alexa Fluor® 488-conjugated and Alexa Fluor® 594 conjugated antibody (1:500, Thermo Fisher, USA) were used as secondary antibodies. The cell nuclei were stained with 40,6-diamidino-2-phenylindole (DAPI) (1:1000, Beyotime, China). Images were acquired under laser confocal microscopy (Leica, Germany) and quantitative analyses of the OCN and IL-1β expression levels were identified using ImageJ.

### Statistical analysis

Experiments were independently repeated three times, with results represented as the mean ± standard deviation. Statistical analysis was performed using SPSS software (SPSS Software, USA). Independent sample t-tests and one-way analysis of variance tests were used to assess differences between groups. A *p*-value < 0.05 was considered statistically significant.

## Results

### PDA/BBR nanoparticle and PDA/BBR@Gel microsphere characterization

**Figure 1:**
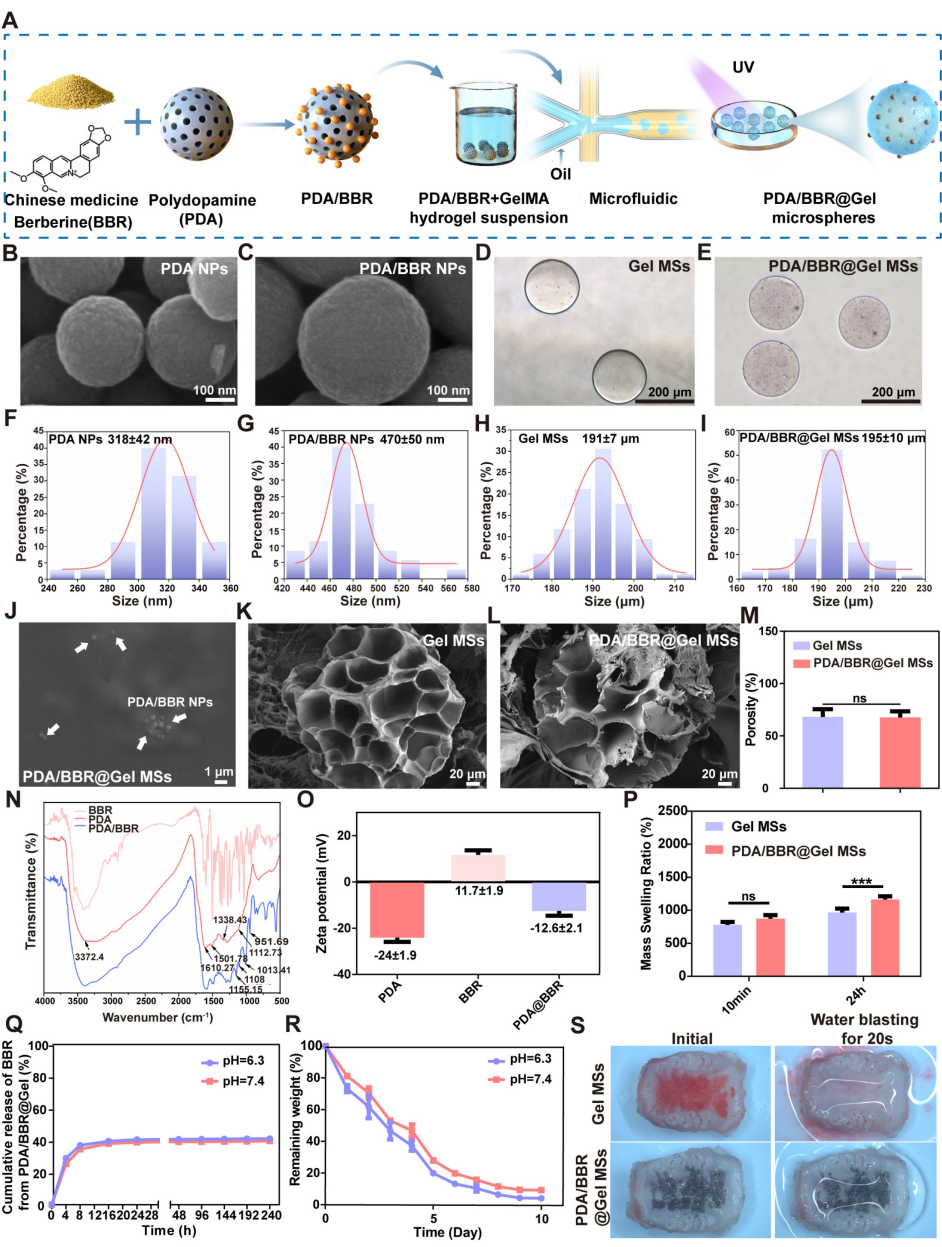
** Physical characteristics of PDA/BBR nanoparticles and PDA/BBR@Gel microspheres** (A) Material preparation process. (B, C) SEM image showing PDA and PDA/BBR nanoparticles. (D) Bright-field image showing pure GelMA microspheres. (E) Bright-field image showing PDA/BBR@Gel microspheres. (F, G, H, I) Diameter distributions of PDA nanoparticles, PDA/BBR nanoparticles, GelMA microspheres, and PDA/BBR@Gel microspheres. (J) PDA/BBR nanoparticles on PDA/BBR@Gel microsphere surfaces. (K, L) SEM images showing the internal structure of GelMA microspheres and PDA/BBR@Gel microspheres. (M) Porosity of GelMA microspheres and PDA/BBR@Gel microspheres. (N) FTIR results for PDA/BBR nanoparticles (O) Zeta potential of various nanoparticles. (P) GelMA microspheres and PDA/BBR@Gel microspheres swelling ratios after soaking for 10 min and 24 h. (Q) Drug release efficiency of PDA/BBR@Gel microspheres at different pH values (6.3 and 7.4). (R) Degradability of PDA/BBR@Gel microspheres at different pH values (6.3 and 7.4). (S) Photographs of the GelMA microspheres and PDA/BBR@Gel microspheres adhered to the isolated rat maxillary gingival tissues before and after water blasting for 20 s. (***p < 0.001, not significant (ns) *p >* 0.05).

The preparation process of PDA nanoparticles used as carriers to improve BBR bioavailability is shown in Figure 1A. SEM images showed that PDA nanoparticles were uniform in size (Figure 1B), with a diameter of 318 ± 42 nm (Figure 1F). However, adding BBR to nanoparticles significantly increased the diameter to 470 ± 50 nm (Figures 1C and 1G). After loading PDA/BBR onto GelMA microspheres (Figure 1D), nanoparticles were dispersed within and on hydrogel microsphere surfaces as observed by optical microscopy (Figure 1E). Importantly, GelMA microspheres were uniform in size at 191 ± 7 μm, and PDA/BBR@Gel microsphere sizes remained stable at 195 ± 10 μm (Figure 1H-I). SEM images further confirmed the successful loading of PDA/BBR nanoparticles within and on the surface of Gel microspheres (Figure 1J and Figure 1L). The porosity of the microspheres was also measured by SEM images. Results showed that the porosity of GelMA microsphere was 68.19% and that of PDA/BBR@Gel microsphere was 67.73%, indicating uniformly dispersed microporous structures conducive to cell growth (Figure 1K-M).

To further confirm the BBR introduction, FTIR analysis was conducted, with spectra showing characteristic absorption peaks in the PDA nanoparticle group at 3372.4 cm (O-H and N-H stretching vibrations), 1610 cm (aromatic ring stretching and N-H bending vibrations), 1501 cm (N-H shearing vibrations), 1338 cm (C-O-H bending and stretching vibrations), and 1112 cm (C-O vibrations). BBR group spectra showed many fine and dense peaks typical of small molecules. In PDA/BBR group spectra, corresponding characteristic peaks were still observed at respective positions, along with characteristic BBR peaks, indicating successful BBR introduction into PDA/BBR nanoparticles (Figure 1N). In addition, the zeta potential assessments were conducted to measure the potential value of PDA, BBR, and PDA/BBR nanoparticles, which changed from -24 mV, 11.7 mV to -12.6 mV (Figure 1O).

GelMA microsphere and PDA/BBR@Gel microsphere hydrophilicity were evaluated by measuring swelling rates in PBS (pH = 7.4). Both samples showed excellent water absorption and expansion effects. At 10 min, no significant differences in swelling rates were observed between samples, but after 24 h, PDA/BBR@Gel microsphere swelling rates were higher than those of GelMA microspheres (Figure 1P). Thus, during soaking processes, PDA/BBR@Gel microspheres absorbed the surrounding liquid much faster than GelMA microspheres, potentially providing better survival conditions for surrounding cells. The mechanical characteristics of the hydrogel media were key factors determining the mechanical performance of microspheres 49. First, we conducted a compressive test on the hydrogel samples. As demonstrated in Figure S1A-B, GelMA and PDA/BBR@GelMA hydrogels could withstand stress between the range of 53-58 kPa, while their corresponding compressive modulus was 13.15 ±1.28 kPa and 22.82 ±0.97 kPa, respectively. Besides, the stability of GelMA and PDA/BBR@GelMA hydrogels was also assessed in the frequency-dependent mode of the rheometer (Figure S1C). The results showed that the storage modulus (G') was consistently larger than the loss modulus (G") when the frequency ranged from 0.1 to 100 rad/s, indicating the stable and elastic performance of the hydrogels.

Next, we investigated the release kinetics and degradation of PDA/BBR@Gel microspheres. We first measured the absorbance of different BBR concentrations to plot a drug concentration versus absorbance standard curve (Figure S2). PDA/BBR@Gel microspheres were immersed in PBS under various pH conditions (pH = 6.3 and 7.4) containing 0.1 U/mL collagenase II at 37 °C, allowing for full BBR release. A steep initial release from PDA/BBR@Gel microspheres was observed in the first 8 hours but became slower and stable over time, indicating good release performance for sustained drug delivery. By the 10th day, the cumulative release ratio reached 40% in a neutral pH environment, while BBR release showed a slight increase to 41% in mildly acidic conditions (Figure 1Q). The degradation performance of PDA/BBR@Gel microspheres *in vitro* was also carried out by placing them in PBS under different pH conditions (pH = 6.3 and 7.4) containing 0.1 U/mL collagenase II at 37 °C. As depicted in Figure 1R, the hydrogel microspheres degraded within 10 days *in vitro* with sustained drug release from PDA/BBR nanoparticles. Besides, the microspheres degraded slightly faster in mildly acidic PBS (pH = 6.3), indicating suitable usages for therapy in the mildly acidic condition of periodontitis (Figure 1R).

Furthermore, we measured the adhesion ability of PDA/BBR@Gel microspheres in the isolated rat maxillary gingival tissues. As shown in Figure 1S and Movies S1 and S2, after placing GelMA microspheres (dyed in red ink) and PDA/BBR@Gel microspheres (dyed in black ink) in the isolated rat maxillary gingival tissues, only PDA/BBR@Gel microspheres could withstand the water blasting for some time, demonstrating strong tissue adhesion properties and significantly strengthening the therapeutic premise of PDA/BBR@Gel microspheres for periodontitis.

### Biocompatibility and antimicrobial performance of PDA/BBR@Gel microspheres

**Figure 2:**
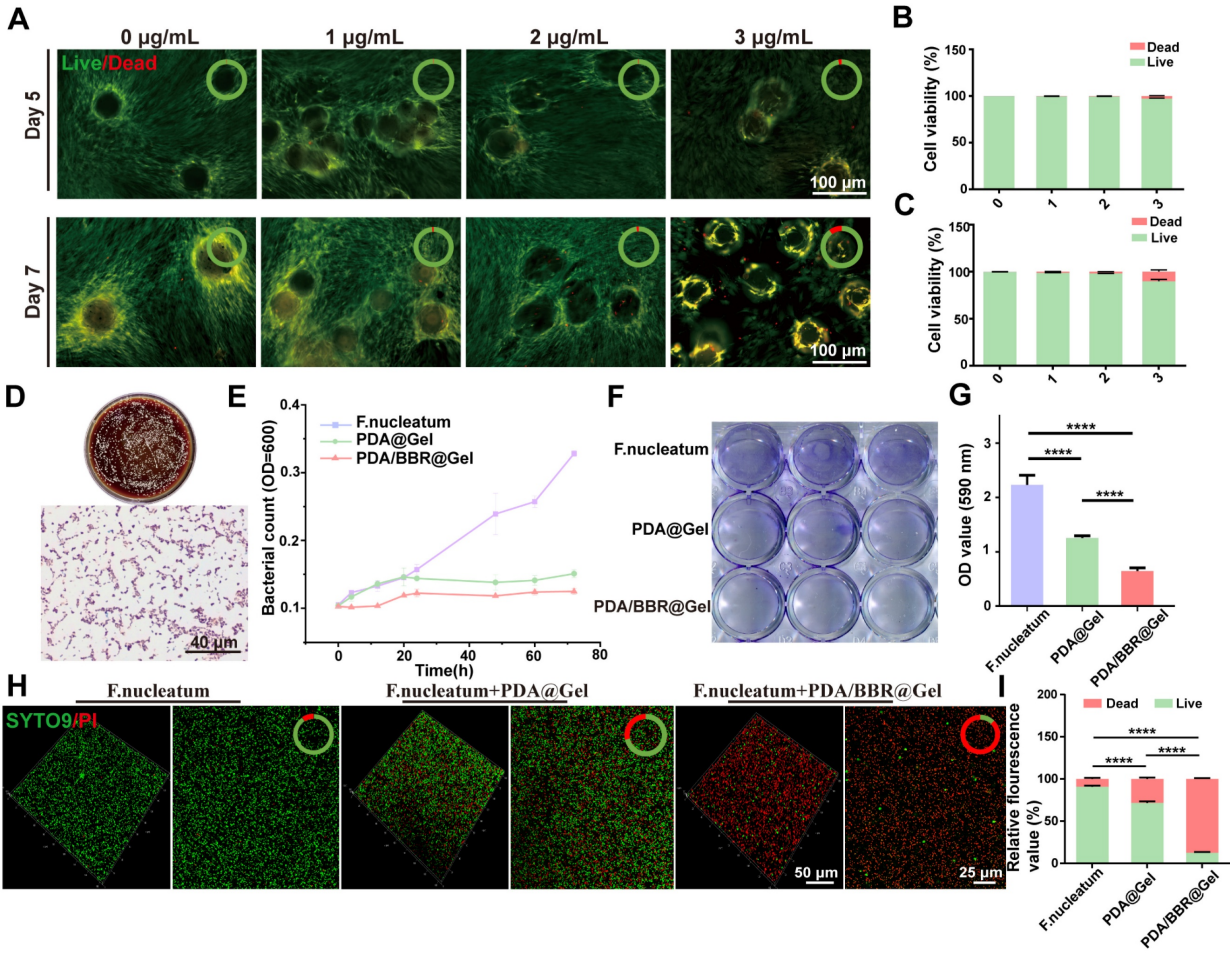
** PDA/BBR@Gel microsphere biocompatibility and antimicrobial performance** (A) Live/dead staining results for PDLSCs after co-culturing with PDA/BBR@Gel microspheres on days 5 and 7. (B, C) Quantitative analysis of live/dead staining on days 5 and 7. (D) *F. nucleatum* colonies on Columbia blood agar plate and Gram staining results. (E) Bacterial growth curve showing *F. nucleatum* co-cultured with PDA/BBR@Gel microspheres for 72 h. (F) Crystal violet staining of *F. nucleatum* biofilms co-cultured with PDA/BBR@Gel microspheres for 72 h. (G) Relative quantitative crystal violet staining results for *F. nucleatum* biofilms co-cultured with PDA/BBR@Gel microspheres for 72 h. (H) SYTO9-PI staining of *F. nucleatum* biofilms co-cultured with PDA/BBR@Gel microspheres for 72 h. (I) Quantitative analysis of SYTO9-PI staining. (****p < 0.0001).

We evaluated PDA/BBR@Gel microsphere biocompatibility and optimized the most appropriate BBR concentration by co-culturing PDLSCs with PDA@Gel microspheres containing various BBR concentrations, followed by live/dead cell staining on days 5 and 7. Live and dead cells fluoresced green and red, respectively (Figure 2A). As BBR concentrations and culture time increased, dead cells increased on microsphere surfaces. On day 5, the 3 μg/mL BBR group had a cell death rate of 6.14% (Figure 2B), which increased to 16.37% on day 7. However, the 2 μg/mL group had a cell death rate of only 7.72% on day 7 (Figure 2C), indicating better cell viability. Thus, 2 μg/mL BBR was selected for further studies.

*F. nucleatum* is the dominant antibiotic-resistant bacterium in periodontitis. To detect its behavioral changes and mechanisms after co-culturing with PDA/BBR@Gel microspheres, *F. nucleatum* was first cultured *in vitro*. Revived *F. nucleatum* grew well, forming irregular circles and slightly convex colonies on Columbia blood agar plates. Gram staining results demonstrated that the revived *F. nucleatum* was Gram-negative and rod-shaped bacteria (Figure 2D). To evaluate PDA/BBR@Gel microsphere inhibitory effects on *F. nucleatum* growth, we monitored growth curves and observed prominent antibacterial effects within 72 h (Figure 2E), demonstrating that PDA/BBR@Gel microspheres exerted long-lasting antibacterial effects by slowly releasing BBR.

Bacterial biofilms enhance bacterial survival and drug resistance, complicating periodontitis treatment. We performed crystal violet and SYTO-9-PI staining to assess biofilm inhibition by PDA/BBR@Gel microspheres. Crystal violet staining showed that biofilm thickness and staining intensity levels were reduced in PDA@Gel and PDA/BBR@Gel microsphere groups when compared to the *F. nucleatum* group. PDA/BBR@Gel microspheres significantly inhibited biofilm growth, even with less residual biofilm (Figure 2F-G). SYTO9-PI staining also revealed significant reductions in biofilm thickness, decreased bacterial counts, and a predominance of dead bacteria in the PDA/BBR@Gel microsphere group, with only a few live bacteria remaining (Figure 2H-I). Thus, PDA/BBR@Gel microspheres effectively inhibited *F. nucleatum* growth and biofilm formation, thereby reducing *F. nucleatum* virulence in periodontitis.

Besides, *P. gingivalis*,* A. actinomycetemcomitans* are also significant pathogenic periodontitis bacteria. To expand the antimicrobial evaluation of PDA/BBR@Gel microspheres, biofilm inhibition assessments of microspheres against *P. gingivalis* and *A. actinomycetemcomitans* were performed with crystal violet staining. Revived *P. gingivalis* and *A. actinomycetemcomitans* grew well and formed colonies with their specific features on Columbia blood agar plates (Figures S3A and S3D). Crystal violet staining showed that biofilm thickness and staining intensity levels of *P. gingivalis* and *A. actinomycetemcomitans* were significantly reduced when treated with PDA/BBR@Gel microspheres (Figures S3B and S3E). Quantitative analysis of crystal violet staining also demonstrated the effective antimicrobial role of PDA/BBR@Gel microspheres against *P. gingivalis* and *A. actinomycetemcomitans* (Figures S3C and S3F).

### Construction of PDA/BBR@Gel@BMP9-PDLSC microspheres

**Figure 3:**
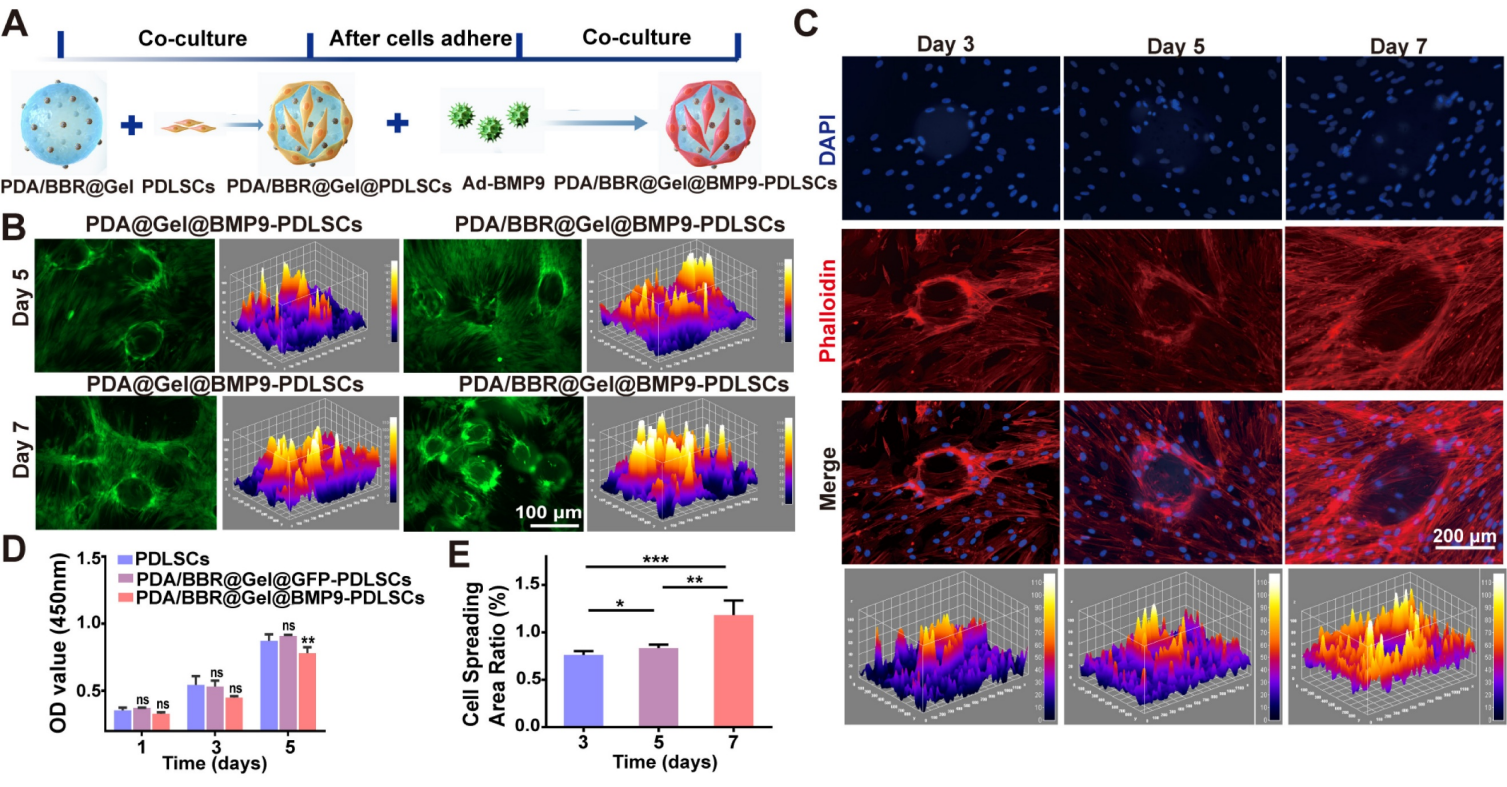
** PDA/BBR@Gel@BMP9-PDLSC microsphere construction** (A) Co-culture formula of PDA/BBR@Gel microspheres, PDLSCs, and Ad-BMP9. (B) GFP images of Ad-BMP9-infected PDLSCs on PDA/BBR@Gel microsphere surfaces. (C) PDLSC phalloidin staining on PDA/BBR@Gel microsphere surfaces on days 3, 5, and 7. (D) PDLSC proliferation curves (CCK-8) on days 1, 3, and 5. (E) Quantified phalloidin staining. (*p < 0.05, **p < 0.01, ***p < 0.001, not significant (ns) *p >* 0.05).

PDA/BBR@Gel microspheres represent three-dimensional (3D) cell culture scaffolds and should offer an outstanding biological microenvironment for cell proliferation and differentiation. Thus, we used PDA/BBR@Gel microspheres to load Ad-BMP9-stimulated PDLSCs, thereby constructing an engineered microsphere system, PDA/BBR@Gel@BMP9-PDLSCs, to secrete BMP9. The experimental procedure showing the culturing of Ad-BMP9-infected PDLSCs on PDA/BBR@Gel microspheres is shown in Figure 3A. The successful loading of Ad-BMP9-infected PDLSCs onto PDA/BBR@Gel microspheres is also shown (Figure 3B), where GFP indicates infected cells. The CCK-8 assay was next used to evaluate the growth and proliferation of Ad-BMP9- infected PDLSCs when co-cultured with PDA/BBR@Gel microspheres. We observed increased cell proliferation over time with no significant differences between groups on days 1 and 3, while cell proliferation rates decreased slightly in the PDA/BBR@Gel@BMP9 microsphere group compared to other groups on day 5 (Figure 3D). This was possibly due to BMP9 addition, which appeared to promote cell differentiation and inhibit cell growth simultaneously. Phalloidin staining was used to assess cell adhesion and spread, showing that Ad-BMP9-infected PDLSCs were significantly increased on microspheres over 3, 5, and 7 days, nearly covering surfaces by day 7 (Figure 3C). Image J analysis confirmed a statistically significant increase in cell-spread area ratios over time (Figure 3E). These data indicated that PDA/BBR@Gel microspheres containing 2 μg/mL of BBR showed good biocompatibility, supporting the normal growth and proliferation of Ad-BMP9-infected PDLSCs, thereby validating the successful construction of PDA/BBR@Gel@BMP9-PDLSC microspheres.

### Osteogenic differentiation and anti-inflammatory actions of PDA/BBR@Gel@BMP9-PDLSC microspheres under *in vitro* inflammatory conditions

While PDA/BBR@Gel microspheres with BBR (2 μg/mL) possessed superior biocompatibility and antimicrobial performance, the osteogenic differentiation of PDLSCs co-cultured with the PDA/BBR@Gel microsphere/BMP9 combination remained unclear. Thus, PDA/BBR@Gel microspheres with different concentrations of BBR (0, 1, 2, and 3 μg/mL) were reevaluated for their osteogenic differentiation effects in PDLSCs. PDA/BBR@Gel microspheres were co-cultured with Ad-BMP9-infected and non-infected PDLSCs for 7 and 21 days, after which alkaline phosphatase (ALP) and alizarin red S (ARS) staining assays were conducted to assess osteogenic differentiation. On day 7, ALP staining/activity levels were highest at 2 μg/mL BBR in both Ad-BMP9-infected and non-infected cells, while Ad-BMP9-infected cells further enhanced osteogenic differentiation effects. Cell growth and osteogenic differentiation ability were significantly inhibited at 3 μg/mL (Figures S4 and S5). On day 21, ARS also indicated that BBR (2 μg/mL) generated the highest calcium deposition and calcified nodule formation levels in combination with BMP9 (Figures S6 and S7). Thus, BBR at 2 μg/mL was the ideal concentration for subsequent BMP9 studies.

**Figure 4:**
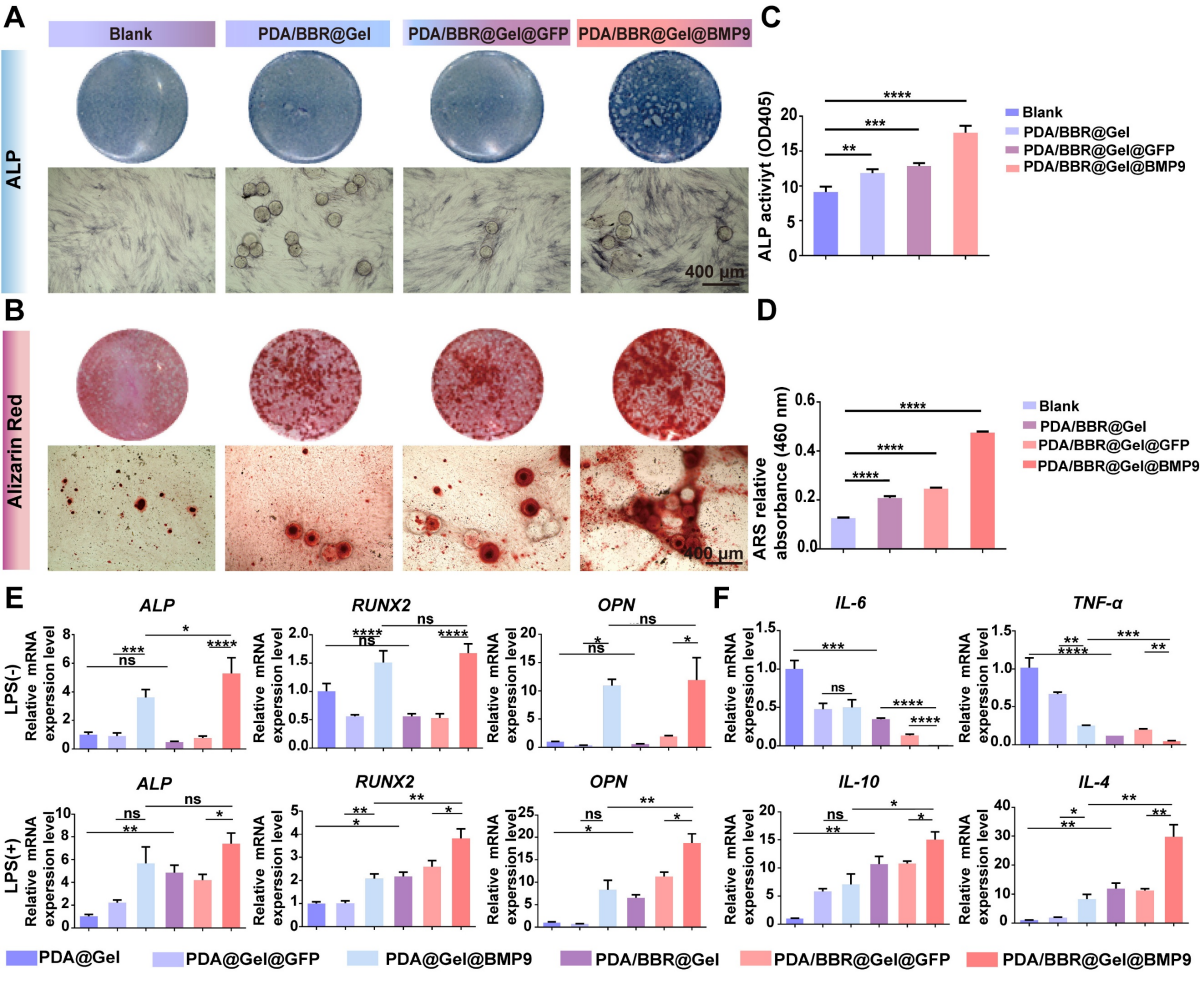
** PDA/BBR@Gel@BMP9-PDLSC microspheres induce osteogenic differentiation and anti-inflammatory effects in an *in vitro* inflammatory environment.** (A) ALP staining of PDLSCs co-cultured with microspheres after 7 days. (B) ARS staining of PDLSCs co-cultured with microspheres after 21 days. (C) ALP quantitation after 7 days of co-culture. (D) ARS quantitation after 21 days of co-culture. (E) Osteogenesis-related gene expression. (F) Inflammation-related gene expression. (*p < 0.05, **p < 0.01, ***p < 0.001, ****p < 0.0001, not significant (ns) *p >* 0.05).

To simulate an inflammatory periodontitis environment, PDLSCs infected with Ad-BMP9 were co-cultured with PDA/BBR@Gel microspheres (2 μg/mL of BBR) in the presence of 10 μg/mL LPS for 7 and 21 days. ALP and ARS assays indicated that PDA/BBR@Gel microspheres, in combination with BMP9, showed the highest osteogenic effects in the inflammatory microenvironment (Figure 4A-D). RT-qPCR showed increased osteogenic (*ALP*, runt-related transcription factor 2 (*RUNX2*), osteopontin (*OPN*), and anti-inflammatory marker expression interleukin 10 (*IL-10*) and *IL-4*, with reduced pro-inflammatory cytokine expression (*IL-6* and tumor necrosis factor α (*TNF-α*)) in PDA/BBR@Gel microspheres in combination with BMP9 (Figure 4E-F). These results suggested synergistic osteogenic and anti-inflammatory effects due to BBR/BMP9 in PDA/BBR@Gel@BMP9-PDLSC microspheres.

### PDA/BBR@Gel@BMP9-PDLSC microspheres synergistically promote cranial bone defect repair

**Figure 5:**
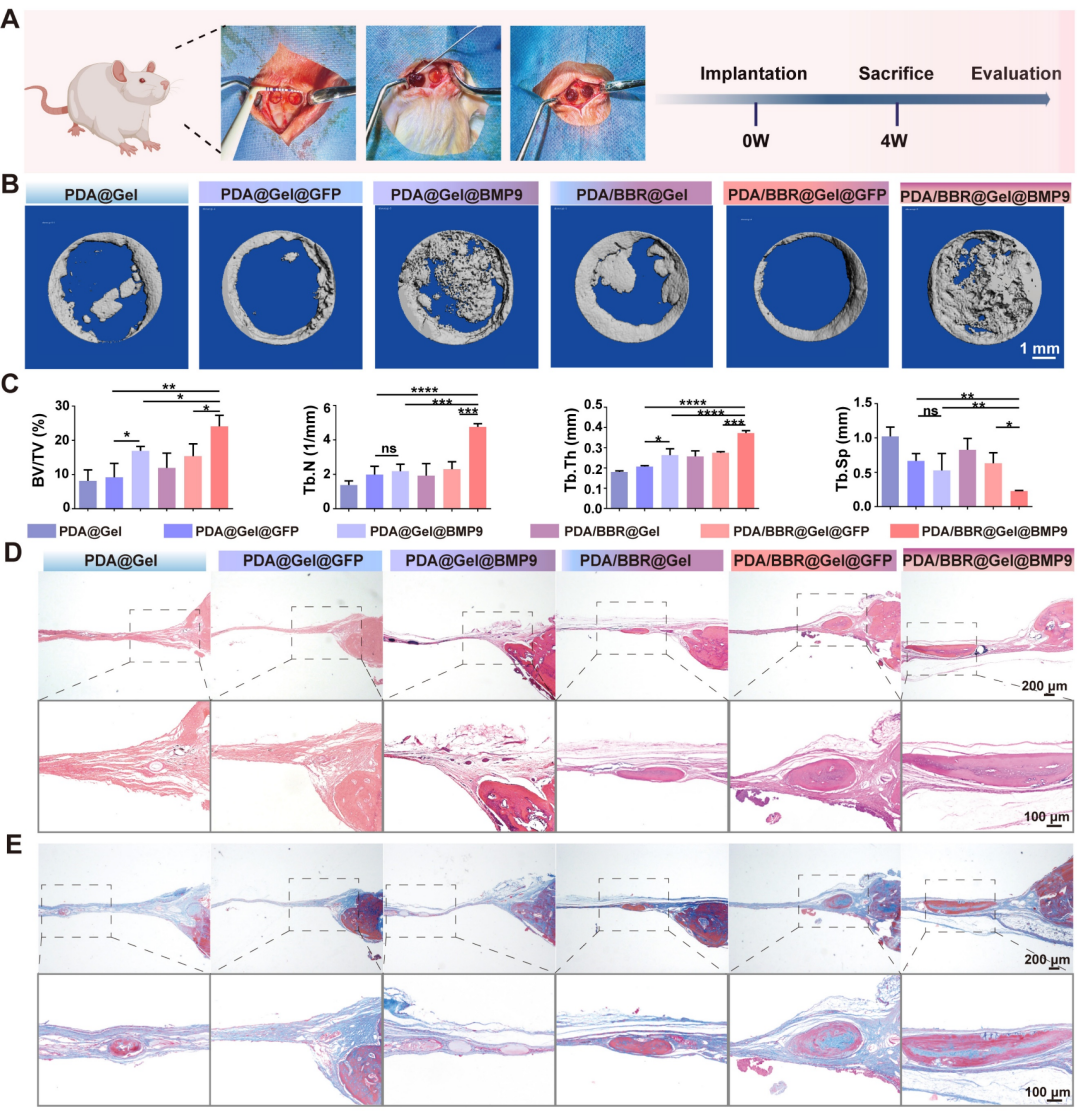
** PDA/BBR@Gel@BMP9-PDLSC microspheres repair cranial defects.** (A) Cranial defect and treatment model in SD rats (Created with BioRender.com). (B) Micro-CT 3D reconstruction images show defect sites after 4 weeks of treatment with different microsphere groups. (C) Micro-CT analysis shows bone volume fraction (BV/TV), number of trabeculae (Th.N), trabecular demarcation thickness (Tb.Th), and trabecular absorption-related indicators. (D) H&E staining shows defect sites after 4 weeks of treatment with different microsphere groups. (E) Masson's trichrome staining shows defect sites after 4 weeks of treatment with different microsphere groups. (*p < 0.05, **p < 0.01, ***p < 0.001, ****p < 0.0001, not significant (ns) *p >* 0.05).

To demonstrate *in vivo* osteogenic effects, a cranial bone defect model was created, and PDA/BBR@Gel@BMP9-PDLSC microspheres were injected into defects. Microspheres adhered well to cranial defects without displacement (Figure 5A). Micro-computed tomography (CT) and histology were used to assess new bone formation after 4 weeks of treatment. The 3D reconstruction of micro-CT images showed that the PDA/BBR@Gel@BMP9-PDLSC group had superior bone repair, with more new bone volume compared to PDA/BBR@Gel@PDLSC and PDA@Gel@BMP9-PDLSC groups. Importantly, PDA/BBR@Gel@BMP9-PDLSC microspheres nearly filled the entire defect area with newly formed hard tissue (Figure 5B).

Micro-CT data also confirmed that the PDA/BBR@Gel@BMP9-PDLSC group had the highest bone volume/total volume (BV/TV), trabecular number (Tb.N), trabecular thickness (Tb.Th), and the lowest trabecular separation (Tb.Sp) (Figure 5C). At week 4, H&E staining showed that most microspheres had degraded, forming new bone tissue in all groups. The PDA/BBR@Gel@BMP9-PDLSC group had the most mature bone tissue, including hard bone plates and Haversian systems (Figure 5D). Masson staining further confirmed extensive fibrous and mineralized new bone in the PDA/BBR@Gel@BMP9-PDLSC group, forming sheet-like connections (Figure 5E). These results suggested that PDA/BBR@Gel@BMP9-PDLSC microspheres robustly enhanced new bone formation, while BMP9/BBR exerted synergistic osteogenic effects to repair cranial defects.

### PDA/BBR@Gel@BMP9-PDLSC microspheres exert therapeutic effects on periodontitis under persistent *F*. *nucleatum* infection

**Figure 6:**
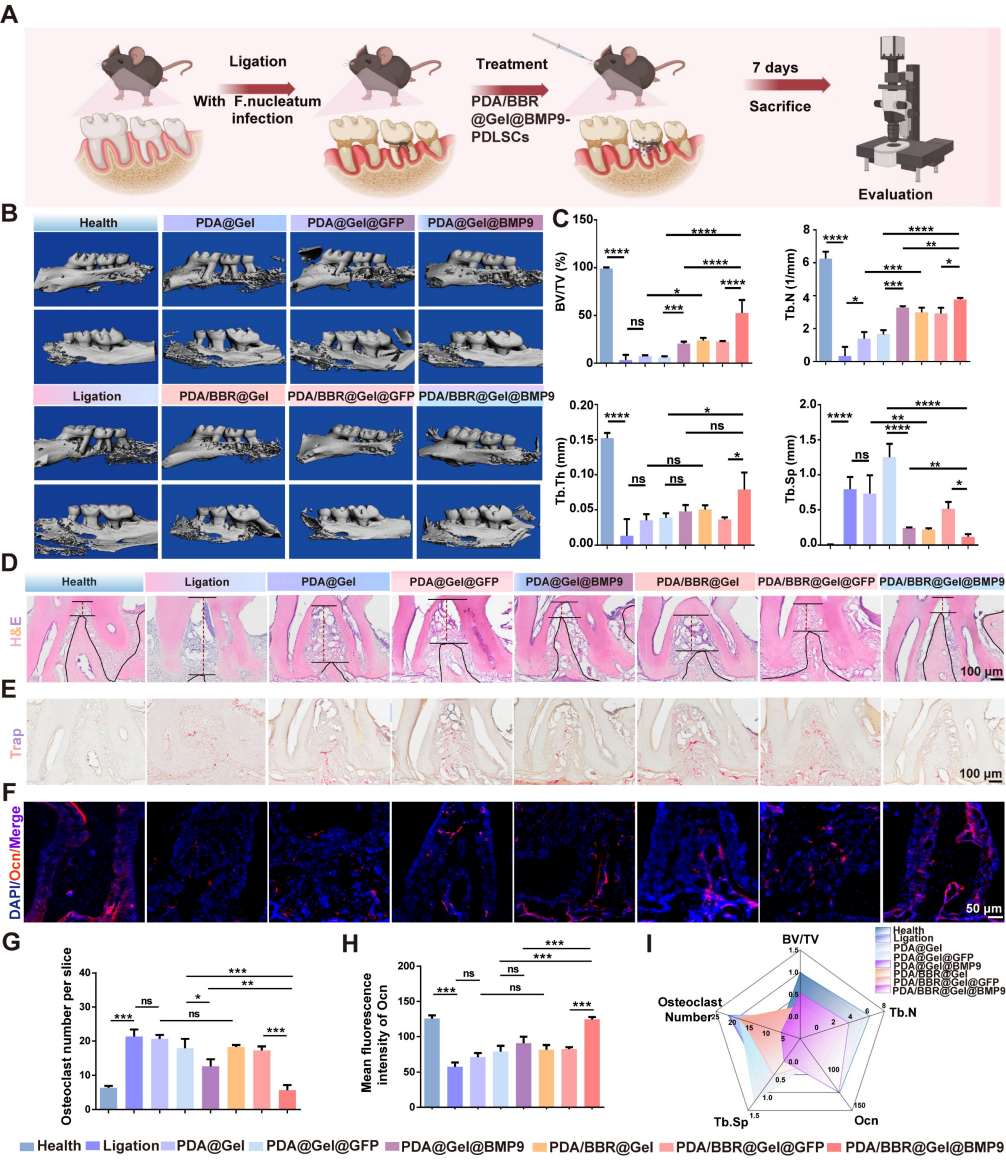
** Effects of PDA/BBR@Gel@BMP9-PDLSC microspheres on periodontitis following persistent *F. nucleatum* infection** (A) Schematic of the experimental procedure (Created with BioRender.com). (B) Micro-CT 3D reconstruction images of the alveolar bone after treatment with different microsphere groups. (C) Micro-CT results of bone volume/total volume (BV/TV), number of trabeculae (Th.N), trabecular demarcation thickness (Tb.Th), and trabecular absorption-related indicators (Tb.Sp). (D) H&E staining of the maxillary alveolar bone sections (E) TRAP staining of the maxillary alveolar bone sections. (F) Immunofluorescence staining of the periodontal tissues showing the Ocn-positive cell distribution (red). Nuclei were stained by DAPI (blue). (G) Quantitative analysis of the TRAP-positive cells per slice. (H) Quantitative analysis of the intensity of Ocn by immunofluorescence. (I) Comprehensive presentation of indicators (BV/TV, Th.N intensity of OCN, Tb. Sp, and osteoclast number) using a radar chart. (*p < 0.05, **p < 0.01, ***p < 0.001, ****p < 0.0001, not significant (ns) *p >* 0.05).

We created a periodontitis model with continuous *F. nucleatum* infection to simulate clinical periodontitis with persistent antibiotic-resistant bacterial infection. Microsphere materials were injected daily into the mouse periodontal pocket. After 7 days, the mouse alveolar bone was removed for micro-CT to assess material effects toward bone regeneration, while periodontal tissues were collected for RT-qPCR to evaluate anti-inflammatory effects (Figure 6A).

The 3D reconstruction of micro-CT images showed severe periodontal bone tissue destruction in the ligature group, with significant buccal and palatal alveolar bone resorption (Figure 6B). Both PDA@Gel@PDLSC and PDA@Gel@GFP-PDLSC microsphere groups showed no significant improvements, while PDA/BBR@Gel@PDLSC and PDA/BBR@Gel@GFP-PDLSC groups exhibited partial bone repair, with some new irregular bone formation. Moreover, PDA@Gel@BMP9-PDLSC and PDA/BBR@Gel@BMP9-PDLSC groups initiated robust alveolar bone healing. Although palatal alveolar bone height in both groups was similar, the PDA/BBR@Gel@BMP9-PDLSC group showed continuous and thicker buccal alveolar bone repair compared to the PDA@Gel@BMP9-PDLSC group, which showed irregular and obvious alveolar bone defects at root apex sites. Micro-CT statistical data analyses were consistent with 3D image reconstruction trends (Figure 6C).

Furthermore, H&E staining was performed to evaluate the periodontium tissue healing quality of different treatment groups at the histological level. Periodontal tissues from healthy mice showed an integrated structure and junctional epithelial attachment that were tightly attached to the enamel margin. However, in the periodontitis group, the junctional epithelial attachment was disrupted, and the height of the alveolar bone was significantly reduced, indicating severe inflammation in the periodontal tissue. In the PDA@Gel@BMP9-PDLSC and PDA/BBR@Gel@GFP-PDLSC groups, the junctional epithelial attachment and alveolar bone height were partially restored compared to the periodontitis group. While in the PDA/BBR@Gel@BMP9-PDLSC group, the periodontium tissues were almost restored and exhibited close to normal morphology, suggesting the most significant remission of inflammation and optimized periodontal health compared to other treated groups (Figure 6D).

We further assessed the anti-osteoclast effect of the material by TRAP staining. The increased number of TRAP-positive (stained red) cells demonstrated an apparent promotion of bone destruction. Many TRAP-positive cells were observed in the ligation group compared to the healthy group. As expected, the TRAP-positive cells in the PDA@Gel@BMP9-PDLSC group were partially reduced. The PDA/BBR@Gel@BMP9-PDLSC group exhibited obvious reduction of TRAP-positive cells and was not significantly different from the healthy group (Figures 6E and 6G). Immunofluorescence staining and quantitative analysis revealed that positive staining for osteocalcin (Ocn) in the PDA/BBR@Gel@BMP9-PDLSC group was notably increased compared to other groups (Figure 6F and Figure 6H). Collectively, the PDA/BBR@Gel@BMP9-PDLSC microspheres were efficient in periodontitis treatment, including the promotion of bone regeneration and reduction of bone loss (Figure 6I).

**Figure 7:**
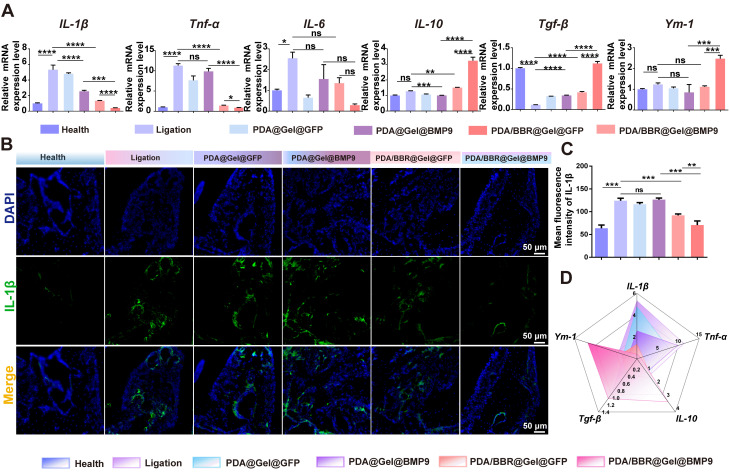
** PDA/BBR@Gel@BMP9-PDLSC microsphere effects on periodontitis with persistent *F. nucleatum* infection** (A) RT-qPCR analysis of gingival tissues after persistent *F. nucleatum* infection (during periodontitis) and treatment using PDA/BBR@Gel@BMP9-PDLSC microspheres. (B) Immunofluorescence staining of the periodontal tissues showing the distribution of IL-1β positive cells (green). Nuclei were stained by DAPI (blue). (C) Quantitative analysis of the intensity of IL-1β by immunofluorescence. (D) Comprehensive presentation of indicators (relative expression of *IL-1β*, *Tnf-α*, *IL-10*,* Tgf-β,* and *Ym-1*) using a radar chart. (*p < 0.05, **p < 0.01, ***p < 0.001, ****p < 0.0001, not significant (ns) *p >* 0.05).

To further confirm the inflammation state of the periodontal microenvironment, RT-qPCR analysis of periodontal tissues after treatments was conducted. The results showed increased pro-inflammatory factor levels (*IL-1β*, *Tnf-α*, and *IL-6*) in the ligation group compared to the healthy group. Treatment with PDA/BBR@Gel@BMP9-PDLSC microspheres significantly reduced these pro-inflammatory factors but increased anti-inflammatory factors (*IL-10*, transforming growth factor β (*Tgf-β*) and *Ym-1*) (Figure 7A). Immunofluorescence staining and quantitative analysis revealed that positive staining for IL1-β in the PDA/BBR@Gel@BMP9-PDLSC group was notably decreased compared to other groups (Figure 7B-C and Figure S8). Therefore, PDA/BBR@Gel@BMP9-PDLSC microspheres effectively repaired alveolar bone defects and exhibited significant anti-inflammatory properties by inhibiting *F. nucleatum* virulence, reducing pro-inflammatory factors, promoting anti-inflammatory factors, and successfully treating periodontitis under persistent *F. nucleatum* infection (Figure 7D).

### PDA/BBR@Gel@BMP9-PDLSC microspheres show no *in vivo* organ toxicity

At 4 weeks after microsphere implantation in animals, H&E staining showed no significant differences in the heart, liver, spleen, lung, and kidney tissue compared to the blank control group (Figure S9). Combined with *in vitro* biosafety test data, PDA/BBR@Gel@BMP9-PDLSC microspheres showed ideal therapeutic effects and a good biosafety profile.

### Synergistic osteogenesis and anti-inflammatory BBR/BMP9 mechanisms in the inflammatory environment

We further elucidated synergistic osteogenesis and anti-inflammatory mechanisms underpinning BMP9/BBR actions in the inflammatory microenvironment. RNA-Seq was used to analyze DEGs in PDLSCs co-cultured with different microsphere groups (PDA@Gel@PDLSC, PDA/BBR@Gel@PDLSC, PDA@Gel@BMP9-PDLSC, and PDA/BBR@Gel@BMP9-PDLSC microspheres for RNA-Seq, control, BBR, B9, and B9-BBR, respectively) under inflammatory conditions for 5 days. Principal component analysis showed good correlations between triplicate samples in the same group and distinct clustering characteristics across different groups (Figure S10A). The gene expression matrix was normalized to remove batch effects, and a box plot was generated for the normalized dataset (Figure S10B). DESeq2 analysis (|log2FC| ≥ 1 and *p* < 0.05) revealed significant gene expression changes compared to the control group with 1767 and 1238 up- and down-regulated genes in the B9-BBR group, 1592 and 1533 up- and down-regulated genes compared to the B9 group, and 2398 and 1671 up- and down-regulated genes compared to the BBR group (Figure S10C).

**Figure 8:**
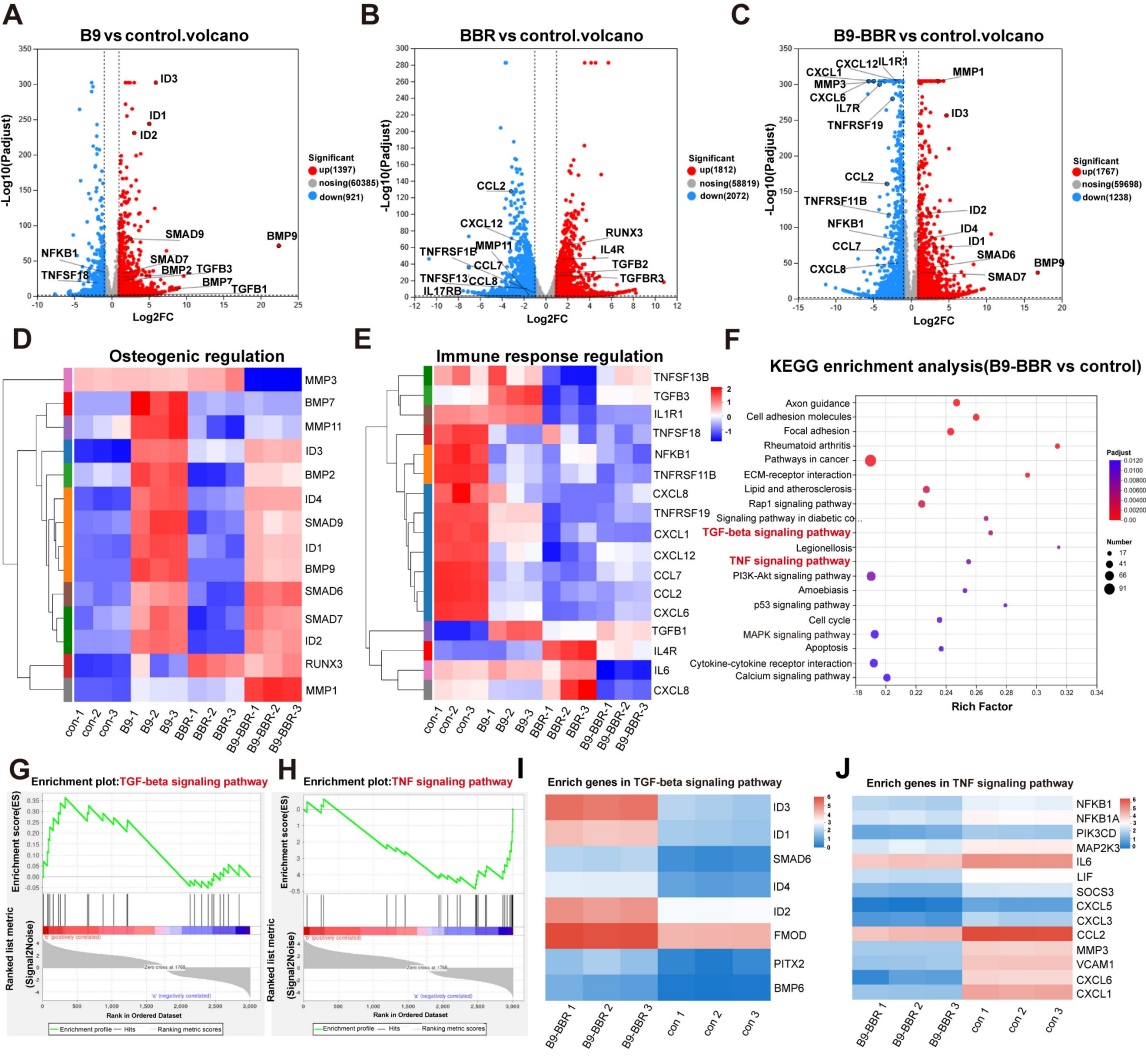
** RNA-Seq differential analysis.** (A, B, C) Volcano plots showing significant differences between groups of differentially expressed genes (DEGs). (D, E) Heatmaps showing DEGs related to osteogenic and immune response regulation. (F) KEGG enrichment analysis of DEGs. (G, H) GSEA plots showing the gene set involved in TGF-β and TNF signaling pathways. (I, J) Heatmaps showing enriched genes in TGF-β and TNF signaling pathways.

A volcano plot showed that DEGs between B9 and control groups were mainly related to osteogenesis, while DEGs between BBR and control groups were primarily associated with immune responses (Figure 8A-B). DEGs between B9-BBR and control groups were related to osteogenesis and immune responses (Figure 8C). Heatmap analyses also showed that key osteogenesis-related genes (*RUNX3, ID2, SMAD7, SMAD6, BMP9, ID1, SMAD9, ID4, BMP2,* and *ID3*) were significantly up-regulated, while immune response genes (*TNFSF13B, IL1R1, TNFSF18, NFKB1, TNFRSF11B, CXCL8, TNFRSF19, CXCL1, CXCL12, CCL7, CCL2, CXCL6, IL6,* and *CXCL8*) were substantially down-regulated in the B9-BBR group (Figure 8D-E).

Furthermore, Kyoto encyclopedia of genes and genomes (KEGG) enrichment analysis was noteworthy due to remarkably enriched TGF-β and TNF signaling pathways relative to immune response regulation and bone reconstruction (Figure 8F). A gene set enrichment analysis (GSEA) plot showed that more genes and stronger associations involving TGF-β signaling were present in the up-regulated fraction of the B9-BBR group compared to the control group. In comparison, more genes involving TNF signaling were present in the down-regulated fraction of the B9-BBR group relative to the control group (Figure 8G-H). We then performed a heatmap analysis of enriched genes in TGF-β and TNF signaling pathways, which showed that compared to the control group, the expression levels of the top enriched genes in the TGF-β signaling pathway, including *ID3, ID1, ID2, SMAD6* and* FMOD*, were significantly up-regulated in the B9-BBR group (Figure 8I). Also, the expression levels of the top enriched genes in the TNF signaling pathway, including *NFKB1, IL6, MMP3* and* CXCL6,* were notably down-regulated in the B9-BBR group (Figure 8J).

**Figure 9:**
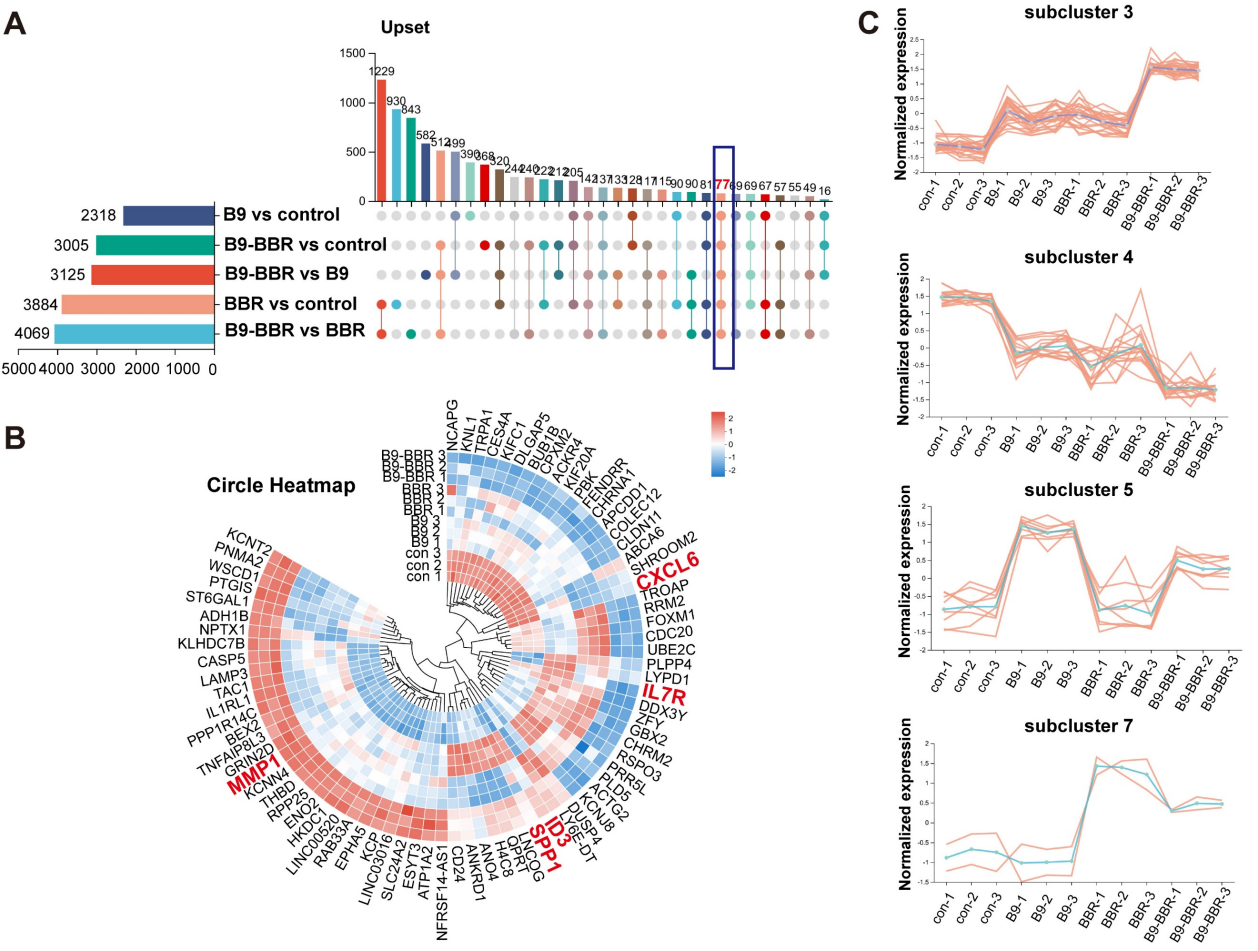
**Analysis of differentially expressed genes and subclusters across groups**. (A) Venn diagram of the commonly differentially expressed genes (DEGs) among groups. (B) Heatmap of common DEGs. (C) DEG subcluster analysis.

We intersected all DEGs across groups to further explore the key factors underpinning these gene expression differences. The Venn diagram analysis identified 77 common DEGs, potentially crucial for the synergistic osteogenesis and anti-inflammatory effects of BMP9 and BBR (Figure 9A). A heatmap was created (Figure 9B) to visualize gene expression differences across groups, after which a subcluster analysis was performed on the 77 common genes. Subcluster 3 genes were increased in both B9 and BBR groups, while the B9-BBR treatment further enhanced gene expression levels. Also, genes in subcluster 4 were decreased in both B9 and BBR groups, while the B9-BBR treatment suppressed gene expression levels. The B9-BBR treatment also increased genes in subclusters 5 and 7, although these genes were only up-regulated in the B9 or BBR groups (Figure 9C). These results indicated the synergistic effects of BMP9 and BBR.

**Figure 10:**
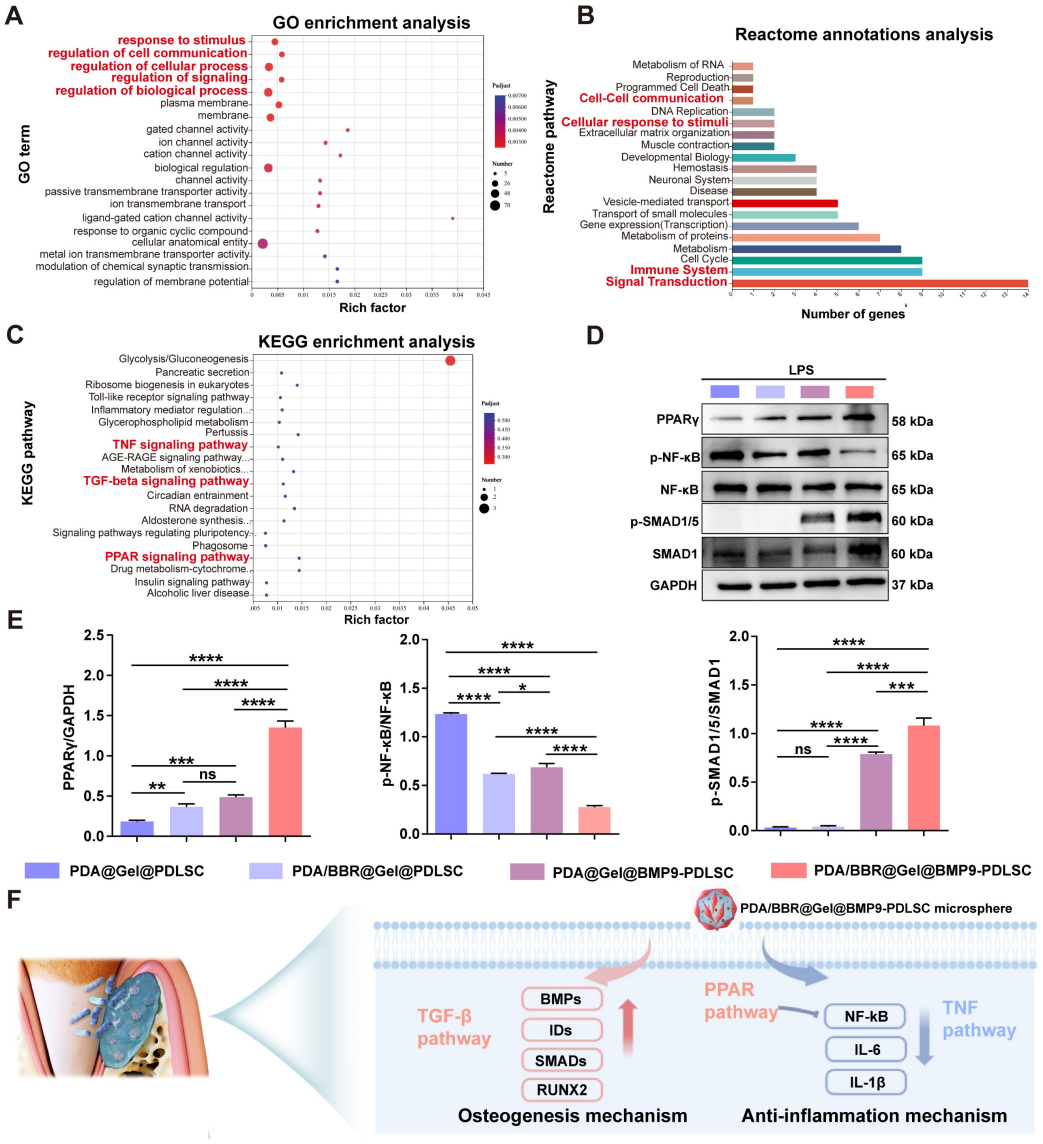
** Genes and pathways involved in regulating synergistic effects of BMP9 and BBR**. (A) Bubble chart of GO enrichment analysis. (B) Reactome annotation analysis. (C) Bubble chart of KEGG enrichment analysis. (D) Western blot analysis of PPARγ, p-NF-κB, NF-κB, p-SMAD1/5, and SMAD1 with GAPDH as the internal control. (E) Semiquantitative analysis of Western blots. (F) Schematic showing the osteogenesis and anti-inflammation mechanism of PDA/BBR@Gel@BMP9-PDLSC microspheres. (*p < 0.05, **p < 0.01, ***p < 0.001, ****p < 0.0001, not significant (ns) *p >* 0.05).

GO enrichment and Reactome annotation analyses revealed that the 77 DEGs were related to cellular responses to stimuli, cell communications, signal transduction, and the immune system (Figure 10A-B). KEGG enrichment analysis also showed that these DEGs were enriched in peroxisome proliferator-activated receptor (PPAR) signaling and TNF and TGF-β signaling (Figure 10C). Thus, PPAR signaling may also be implicated in the synergistic effects of BMP9/BBR. Our RT-qPCR results demonstrated that PDA/BBR@Gel@BMP9-PDLSC microspheres significantly downregulated the mRNA expression level of pro-inflammatory genes (*TNF-α*, *IL-1β,* and *IL-6*) and upregulated the mRNA expression level of anti-inflammatory genes (*IL-10* and *TGF-β*) and osteogenesis-related genes (*ALP*, *RUNX2* and *OPN*) (Figure 4E-F and Figure 7A). Combining these findings with the RNA-seq results, we hypothesize that BBR and BMP9 in microspheres may synergistically inhibit the TNF/NF-κB pathway and promote PPARγ pathway and TGF-β/SMAD pathways under inflammatory conditions to coordinate bone remodeling and immune regulation.

To verify this hypothesis, we evaluated the protein expression levels of PPARγ, p-NF-κB, NF-κB, p-SMAD1/5, and SMAD1 by Western blotting. The results showed that the PPARγ protein expression was upregulated and the p-NF-κB/NF-κB ratio was decreased in PDA/BBR@Gel@PDLSC and PDA@Gel@BMP9-PDLSC groups. At the same time, PDA/BBR@Gel@BMP9-PDLSC microsphere treatment further amplified this trend, demonstrating significant activation of the PPARγ pathway and inhibition of the TNF/NF-κB pathway for anti-inflammatory effects. Besides, the ratio of p-SMAD1/5/SMAD1 was elevated in the PDA@Gel@BMP9-PDLSC group; the PDA/BBR@Gel@BMP9-PDLSC microsphere treatment further amplified this trend, indicating the activation of the TGF-β/SMAD pathway, thereby exerting robust osteogenic effects (Figure 10D-E). In summary, the synergistic osteogenesis and anti-inflammatory mechanisms underpin BBR/BMP9 actions in the inflammatory environment illustrated in Figure 10F.

## Discussion

Considering the irregularity of periodontal tissue defects and the challenges facing clinical treatments, we proposed a novel method using microfluidics to construct microspheres with PDA nanoparticles loaded with BBR, GelMA hydrogel, and Ad-BMP9-infected PDLSCs. PDA/BBR@Gel@BMP9-PDLSC microspheres promoted osteogenic differentiation and displayed considerable antimicrobial and anti-inflammatory abilities under *in vitro* inflammatory conditions. Furthermore, *in vivo* studies indicated that these microspheres promoted cranial bone defect repair and exerted therapeutic effects on periodontitis under persistent *F. nucleatum* infection.

We first successfully fabricated uniformly sized microspheres, which showed good biocompatibility and were minimally invasive when injected into defect sites. Importantly, we exploited microfluidics, suitable GelMA hydrogel characteristics, and excellent PDA nanoparticle properties to create these microspheres 50-54. Microfluidics facilitated successful GelMA hydrogel microsphere synthesis and enabled precise control and large-scale production of microspheres at 195 ± 10 μm dimensions, which were injectable using syringes 55, 56. The 10% concentration of GelMA hydrogel used in our study combined suitable swelling and mechanical properties, potentially providing better survival conditions for stem cells 57, 58. PDA/BBR nanoparticles were innovatively added to the GelMA gel interior and surface, achieving stable and slow drug release and enhancing the wet adhesive property. Also, the common antibiotic resistance issues in clinical practice were avoided through the natural components of Chinese herbal medicine 59.

Furthermore, the appropriate degradation rate of hydrogel microspheres is significant for stem cell migration and controlled drug release to remodel the inflammatory microenvironment. In our study, the mildly acidic condition slightly increased the degradation of hydrogel microspheres, with PDA/BBR nanoparticles providing sustained drug release for remodeling and maintaining the microenvironment in periodontitis. Although only minimal degradation of PDA nanoparticles was observed in the *in vitro* experiments of this study, the microenvironment of periodontitis involves various immune cells, playing an essential role in the biodegradation of PDA nanoparticles 60, 61. In particular, PDA is a well-recognized biopolymer with high degradation characteristics, which can consume excess reactive oxygen species from immune cells at the infection site and be simultaneously oxidized and degraded to soluble PDA oligomers, indicating the biodegradability of PDA* in vivo* 62, 63. However, the detailed degradation mechanism of PDA nanoparticles *in vivo* still needs further exploration.

To enhance microsphere osteogenic ability, Ad-BMP9-infected PDLSCs were seeded onto microsphere surfaces to secrete BMP9, facilitating osteogenic differentiation. Adenoviruses have high transfection efficiencies and persistent gene expression, which allow for stable BMP9 expression in target cells for specific periods and also ensure continuous growth factor release 45, 64. This constant release was crucial for the long-term effects on bone tissue regeneration and repair. Additionally, Ad-BMP9 (expressing GFP) was used to stimulate PDLSCs and then seeding them onto microsphere surfaces, which generated sound material localization and specificity features. As PDLSCs have specific cellular characteristics and biological functions in periodontal tissue, seeding cells stimulated with Ad-BMP9 on microsphere surfaces may facilitate local release and growth factor actions in periodontal tissues, thereby improving the efficiency and accuracy of bone tissue regeneration 65.

Inspired by the hierarchical structure of the lotus (including seedpod, seeds, and flower) and the symbiotic relationship, we developed a nano-micron combined hydrogel microsphere system integrating GelMA microspheres, PDA/BBR nanoparticles, and Ad-BMP9-infected PDLSCs. Specifically, GelMA microspheres mimicking a lotus seedpod acted as a significant intermediary. The synergistic relationship between lotus seeds and flowers accorded them the name “dual-life flower” by the Chinese. Also, it offered valuable inspiration for building high-performance microspheres, possessing superior anti-inflammatory and osteogenic effects with the unique interplay between BBR and BMP9 from PDA/BBR@Gel@BMP9-PDLSC microspheres.

Our antibacterial studies showed that PDA/BBR@Gel microspheres effectively inhibited *F. nucleatum* growth, especially after 72 h of co-culture, indicating the ability to inhibit bacterial proliferation over the long term, potentially attributable to slow and continuous BBR release. Bacterial biofilm formation is closely related to chronic infection and drug resistance, but effective antibacterial materials block this formation 66-68. PDA/BBR@Gel microspheres significantly reduced the biofilm thickness of *F. nucleatum*, *P.gingivalis,* and* A. actinomycetemcomitans*, effectively inhibiting bacterial virulence. Previous studies have revealed that BBR could interfere with bacterial cell wall biosynthesis by inhibiting key enzymes and destroying the bacterial cell membrane integrity 69. It suggested that BBR may suppress the synthesis of critical proteins for bacterial growth and inhibit bacterial metabolism 70. Despite PDA/BBR@Gel microspheres showing potential antibacterial performances, the specific and complex mechanism by which these microspheres act on different bacteria still requires further investigation through multi-omics approaches.

Although many studies have reported that GelMA microspheres alone exerted satisfactory osteogenic effects as scaffold materials in tissue engineering, we showed that BBR/BMP9 introduction enhanced these effects, particularly BBR at 2 μg/mL. We posit the following explanations for why osteogenic effects decreased when the BBR concentration increased to 3 μg/mL. On one hand, high BBR concentrations may exert cytotoxic effects, induce apoptosis, halt cell cycle progression, and even damage cell membranes. Previously, BBR-related cell cycle inhibitory effects were observed in the 30 μM concentration range 71. On the other hand, infection with Ad-BMP9 caused slower cell growth and proliferation, shifting the focus to osteogenic differentiation 72. A notable reduction in cell counts was observed at the 3 μg/mL BBR concentration, accompanied by a slowdown in cell growth. Consequently, this diminished the detection of both ALP and calcified nodules.

Further* in vivo* studies using our periodontitis model with persistent *F. nucleatum* infection revealed that PDA/BBR@Gel@BMP9-PDLSC microspheres were effective as they promoted new bone formation and exhibited significant anti-inflammatory properties. These effects were potentially caused by microsphere spherical structures and surface porosity, which enhanced nutrient uptake and new tissue formation, while continuous BBR/BMP9 release improved the periodontal microenvironment 73-75. The microspheres also inhibited the dominant antibiotic-resistant bacteria (*F. nucleatum*) in periodontitis, promoted osteogenic differentiation of PDLSCs, and decreased the expression levels of inflammatory factors.

Overall, our comprehensive comparative analyses *in vivo* and *in vitro* showed superior osteogenic and anti-inflammatory effects of PDA/BBR@Gel@BMP9-PDLSC microspheres compared to PDA/BBR@Gel@PDLSC and PDA@Gel@BMP9-PDLSC microspheres. We speculate that this may be attributed to BBR/BMP9 synergistic effects in promoting osteogenesis and anti-inflammation processes, which were confirmed by RNA-Seq and Western blotting analyses. The synergy of BMP9 and BBR mainly up-regulated *ID3, ID1, MMP1,* and* SMAD6* genes while down-regulating *IL6, NFKB1, CXCL6,* and* IL-7R* genes, which primarily participated in TGF-β, TNF, and PPAR signaling pathways 76-79. As previous studies have demonstrated, the activation of TGF-β signaling is significant for bone remodeling and formation through TGF-β/BMP/SMADs/RUNX2 pathway 80. Activation of the PPAR signaling pathway and inhibition of the TNF signaling pathway can significantly suppress the NF-κB pathway, which is crucial for the anti-inflammatory process in periodontitis 81, 82. Significantly, our Western blot results are consistent with these studies. In addition, semiquantitative Western blot analysis demonstrated that PDA/BBR@Gel@BMP9-PDLSC microspheres most effectively activated the TGF-β and PPAR pathways while inhibiting the TNF pathway, compared to single-component counterparts (PDA/BBR@Gel@PDLSC and PDA@Gel@BMP9-PDLSC). This enhanced performance may result from their unique interactions, which synergistically amplify the osteogenic and anti-inflammatory effects of the two components. Moreover, we previously identified* ID1*, *ID2,* and *ID3* as the early response target genes of BMP9-mediated osteogenic process 83, 84, which could further confirm the accuracy of the RNA-Seq results in this study.

Therefore, PDA/BBR@Gel@BMP9-PDLSC microspheres effectively treated periodontitis by promoting osteogenesis, reducing inflammation, and overcoming clinical treatment challenges due to the synergistic actions of traditional drugs BBR and BMP9. Our approach holds promise for clinical microsphere applications in comprehensively managing irregular bone defects and persistent inflammation in periodontitis. However, in addition to the osteogenic differentiation of stem cells, the roles of various immune cells involved in the periodontal microenvironment are also essential for the progression of periodontitis. Thus, further investigations are necessary to elucidate the immunomodulatory mechanisms of PDA/BBR@Gel@BMP9-PDLSC microspheres on immune cells within the periodontal inflammatory microenvironment. Besides, a recent study reporting that tilapia fish gelatin had the intrinsic anti-inflammatory activity to regulate the M2 polarization of macrophages 57, provides inspiration for the potential benefit of future research to modify PDA/BBR@Gel@BMP9-PDLSC microspheres further.

## Conclusions

Inspired by the structure of the lotus flower, we successfully used microfluidics to prepare novel GelMA hydrogel microspheres equipped with PDA/BBR nanoparticles and Ad-BMP9-stimulated PDLSCs. These microspheres exhibited excellent biocompatibility, stable drug release, and potent antibacterial properties against *F. nucleatum*. The microspheres significantly reduced inflammation and promoted osteogenic differentiation both* in vitro* and *in vivo*, demonstrating considerable potential for treating periodontitis. We also characterized synergistic mechanisms of BBR and BMP9 in enhancing bone formation and suppressing inflammation via several key genes involved in TGF-β, TNF, and PPAR signaling pathways. PDA/BBR@Gel@BMP9-PDLSC microspheres provide a promising new therapeutic approach for periodontitis, combining modern biomaterial technology with TCM and traditional drugs BBR and BMP9. This integration could provide more effective and safer treatment options, opening new research directions in dental medicine.

## Supplementary Material

Supplementary figures and movies.

## Abbreviations

F. nucleatum: Fusobacterium nucleatum
P. gingivalis: Porphyromonas gingivalis
A. actinomycetemcomitans: Aggregatibacter Actinomycetemcomitans
TCM: Traditional Chinese medicine
BBR: berberine
PDA: polydopamine
BMP9: bone morphogenetic protein 9
PDLSCs: periodontal ligament stem cells
GelMA: gelatin methacrylate
RNA-seq: RNA sequencing
SEM: scanning electron microscopy
FTIR: fourier-transform infrared spectroscopy
PBS: phosphate buffered saline
MEM: modified eagle medium
FBS: fetal bovine serum
LPS: lipopolysaccharide
Ad-GFP: adenovirus with green fluorescent protein
CCK-8: cell counting kit-8
DAPI: 4',6-diamidino-2-phenylindole
ALP: alkaline phosphatase
BCIP: 5-Bromo-4-chloro-3-indolyl phosphate
NBT: nitrotetrazolium blue chloride
ARS: alizarin red staining
BHI: brain-heart infusion broth
DEG: differential gene expression
GO: gene ontology
KEGG: kyoto encyclopedia of genes and genomes
SD: Sprague Dawley
3D: three-dimensional
ROI: region of interest
BV/TV: bone volume/ total volume
Tb.N: trabecular number
Tb.Th: trabecular thickness
Tb.Sp: trabecular separation
EDTA: ethylene diamine tetra acetic acid
H&E: hematoxylin & eosin
TRAP: tartrate-resistant acid phosphatase
Runx2: runt-related transcription factor 2
OPN: osteopontin
IL-10: interleukin 10
TNF-α: tumor necrosis factor α
GSEA: gene set enrichment analysis
PPAR: peroxisome proliferators-activated receptor
