# Supplementary material for: Bioinspired nano-micron hydrogel microspheres for periodontitis therapy through synergistic multi-targeted remodeling of microenvironment
Source: Theranostics. 2025 Jun 9;15(14):6857–81. doi: 10.7150/thno.112782 (PMC12203809; doi:10.7150/thno.112782)
Supplement: Supplementary file 1 — Supplementary figures and movies. [file thnov15p6857s1.zip › Supplemental figures/Supplementary information.pdf]

SUPPORTING INFORMATION

Bioinspired nano-micron hydrogel microspheres for periodontitis therapy through synergistic multi-targeted remodeling of microenvironment

Siqi Zhou[1,2#], Yuxin Zhang[1,2,3#], Liwen Zheng[1,2,3#], Chang Liu[1,2], Jiajun Chen[1,2], Yaxian Liu[1,2], Shidian Ran[1,2], Tong-Chuan He[5], Mengqin Gu[1,4], Si Wu[1,2,4], Fugui Zhang[1], Hongmei Zhang[1,2,4]*

[1] Chongqing Key Laboratory of Oral Diseases, The Affiliated Hospital of Stomatology, Chongqing Medical University, Chongqing, China

[2] Department of Pediatric Dentistry, The Affiliated Hospital of Stomatology, Chongqing Medical University, Chongqing, China

[3] Chongqing Municipal Key Laboratory of Oral Biomedical Engineering of Higher Education, Chongqing, China

[4] Chongqing Municipal Health Commission Key Laboratory of Oral Biomedical Engineering, Chongqing, China

[5] Molecular Oncology Laboratory, Department of Orthopaedic Surgery and Rehabilitation Medicine, The University of Chicago Medical Center, Chicago, Illinois, USA

[#] These authors contributed equally to this work.

* Corresponding author

Correspondence

Hongmei Zhang, DDS, PhD

Department of Pediatric Dentistry

The Affiliated Hospital of Stomatology

Chongqing Medical University

426 Songshibei Road, Chongqing 401147, China

Email: hmzhang@hospital.cqmu.edu.cn

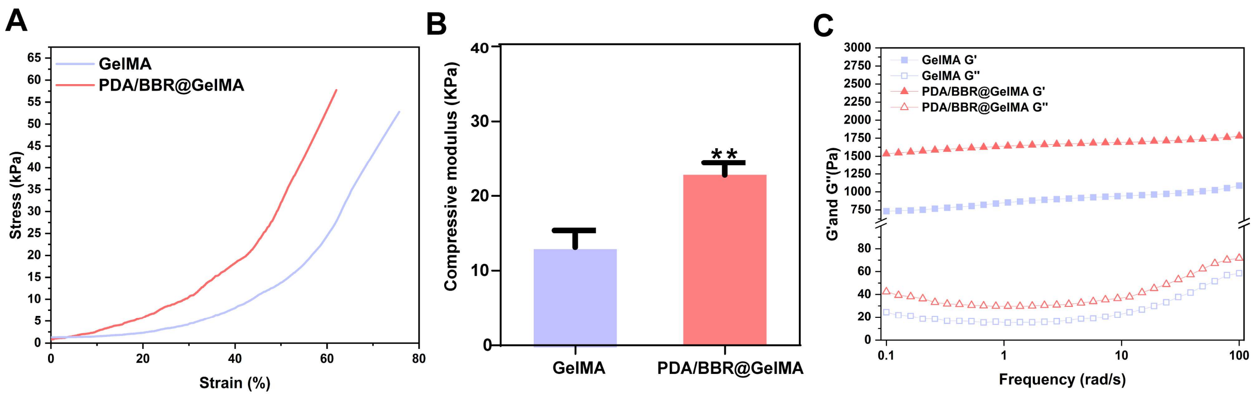

Figure S1. Mechanical properties of GelMA and PDA/BBR@GelMA hydrogels. (A) Representative compressive stress-strain curves. (B) Compressive modulus of the hydrogels (n=3). (C) Storage modulus (G') and loss modulus (G'') between 0.1-100 angular frequencies.

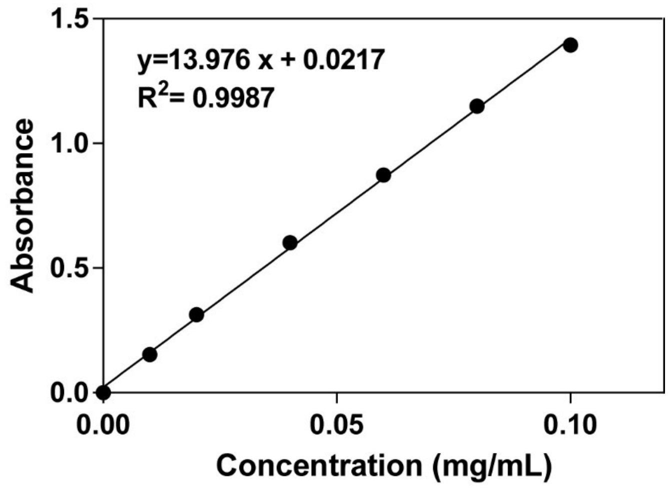

Figure S2. Standard curve of absorbance and concentration of BBR.

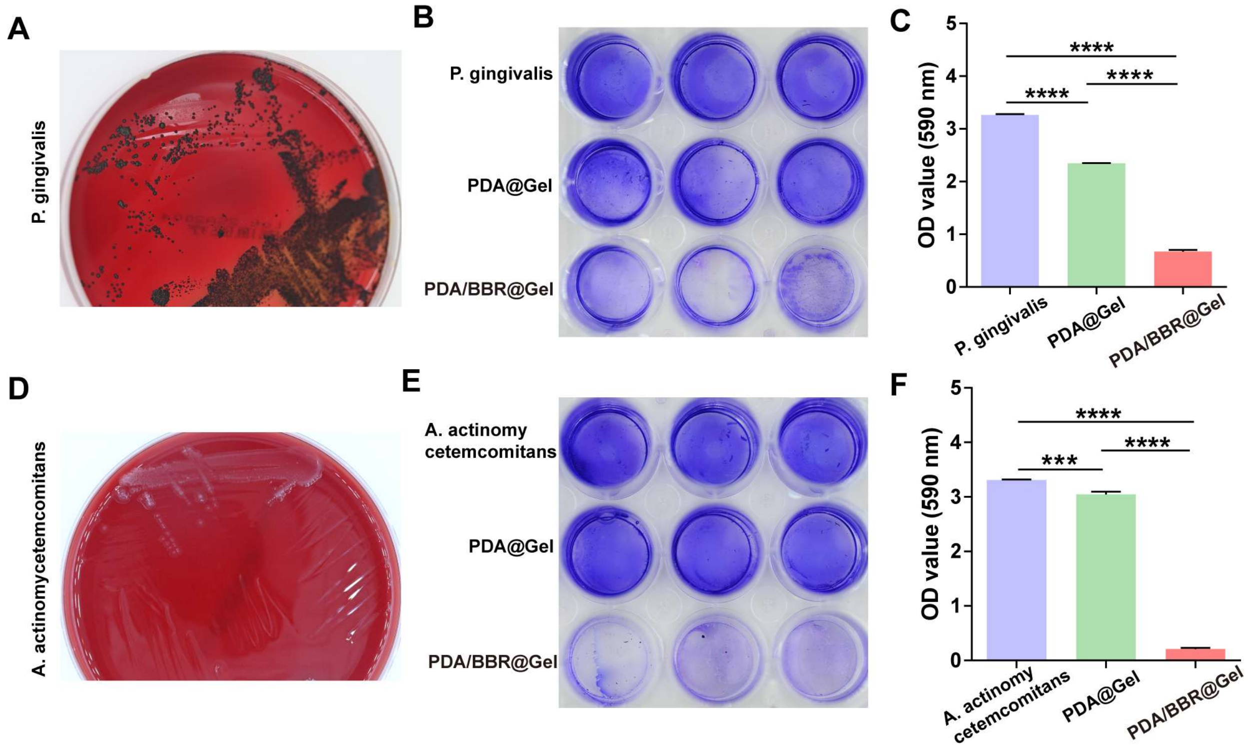

Figure S3. (A) *P. gingivalis* colonies on Columbia blood agar plate. (B) Crystal violet staining of *P. gingivalis* biofilms co-cultured with PDA/BBR@Gel microspheres for 5 days. (C) Relative quantitative crystal violet staining results for *P. gingivalis* biofilms co-cultured with PDA/BBR@Gel microspheres for 5 days. (D) *A. actinomycetemcomitans* colonies on Columbia blood agar plate. (E) Crystal violet staining of *A. actinomycetemcomitans* biofilms co-cultured with PDA/BBR@Gel microspheres for 3 days. (F) Relative quantitative crystal violet staining results for *A. actinomycetemcomitans* biofilms co-cultured with PDA/BBR@Gel microspheres for 3 days. (***$p < 0.001$, ****$p < 0.0001$)

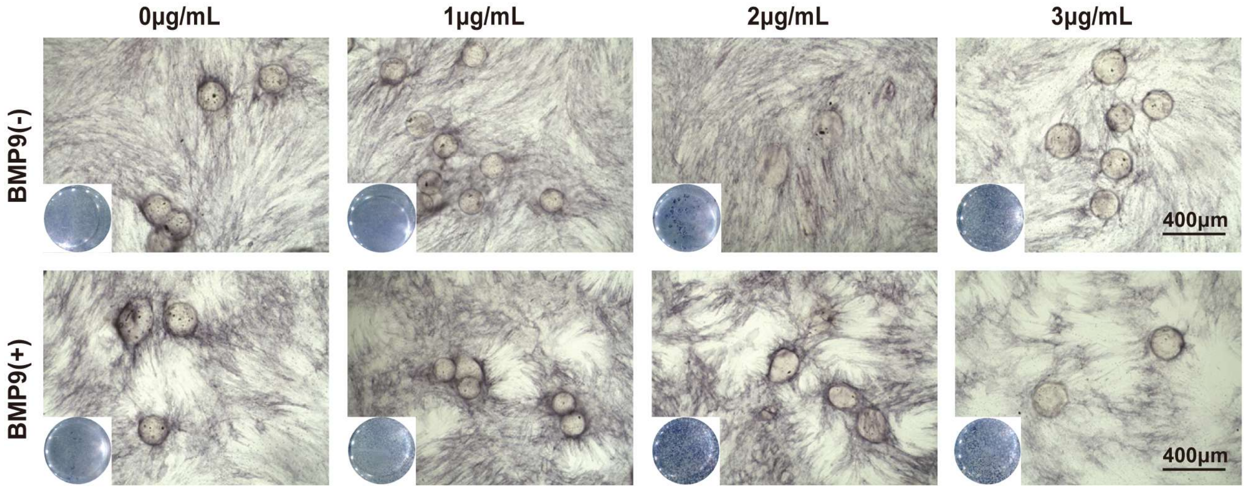

Figure S4. The results of ALP staining after 7 days of co-culture.

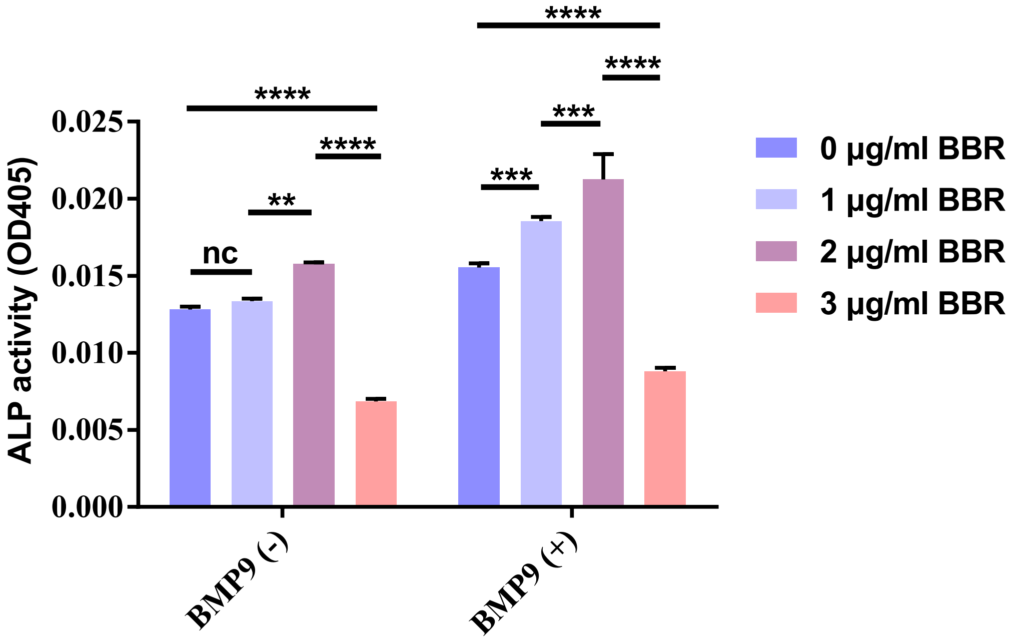

Figure S5. Quantitative results of ALP activity assay after 7 days of co-culture.

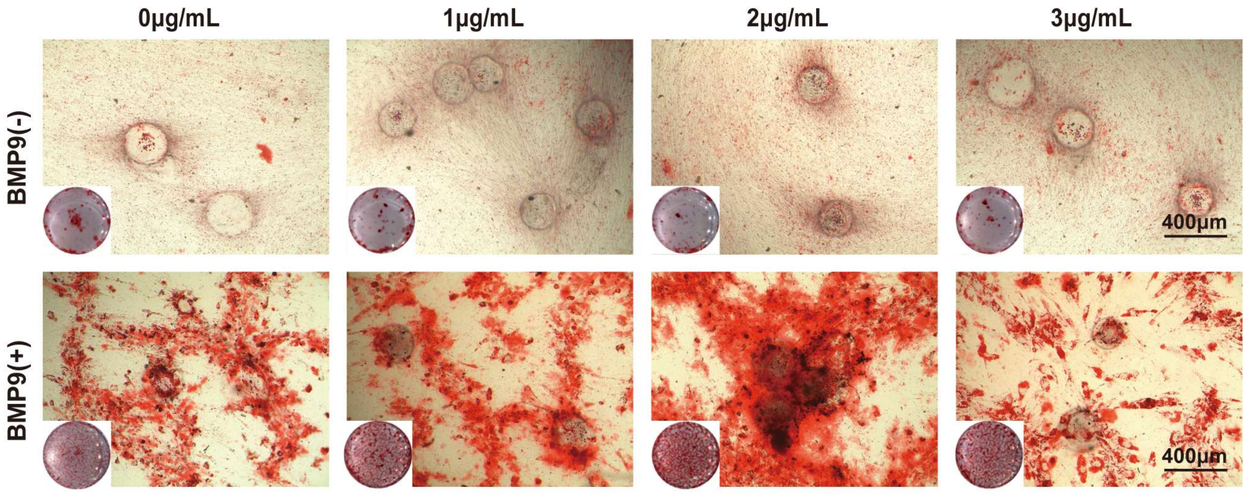

Figure S6. The results of alizarin red staining after 21 days of co-culture.

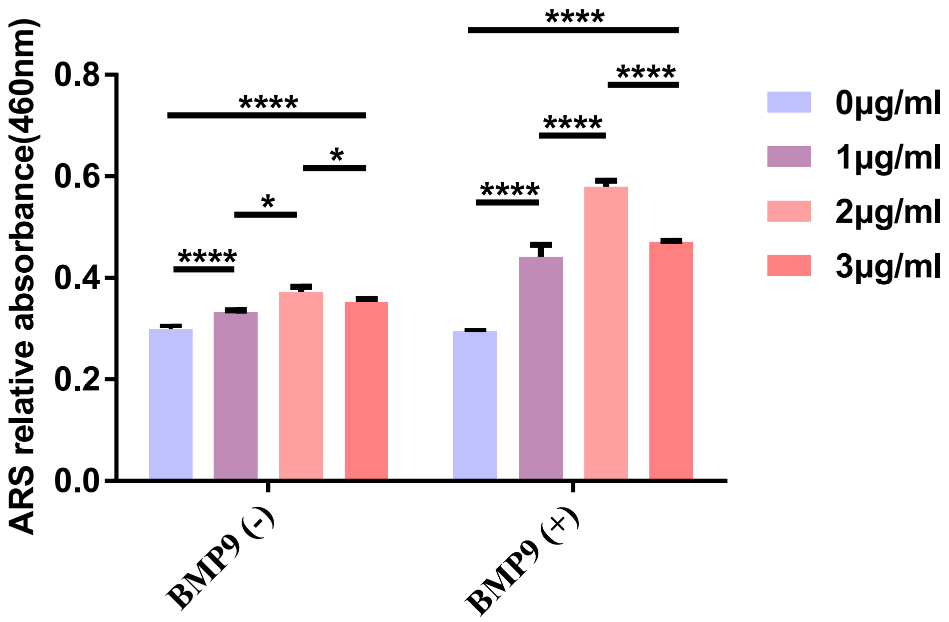

Figure S7. Quantitative results of alizarin red after 21 days of co-culture.

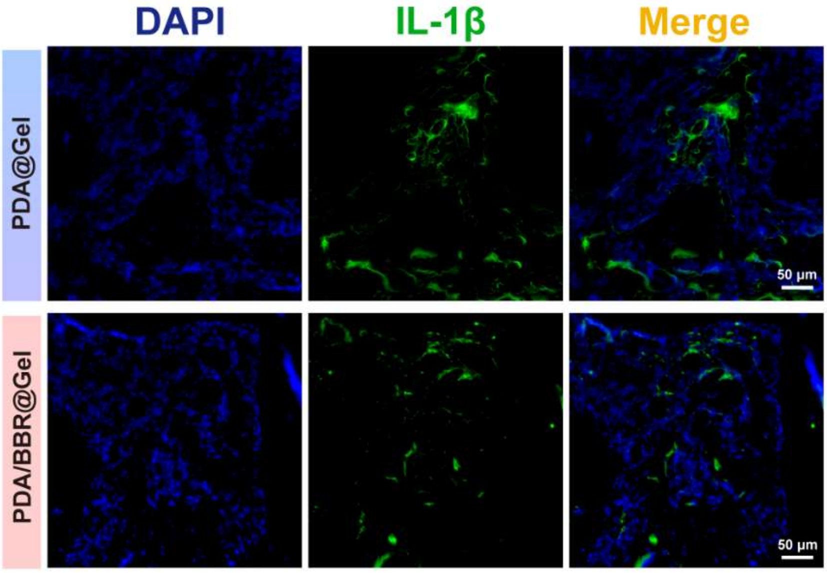

Figure S8. Immunofluorescence staining of the periodontal tissues, showed the distribution of IL-1β positive cells (green). Nucleus were stained by DAPI (blue).

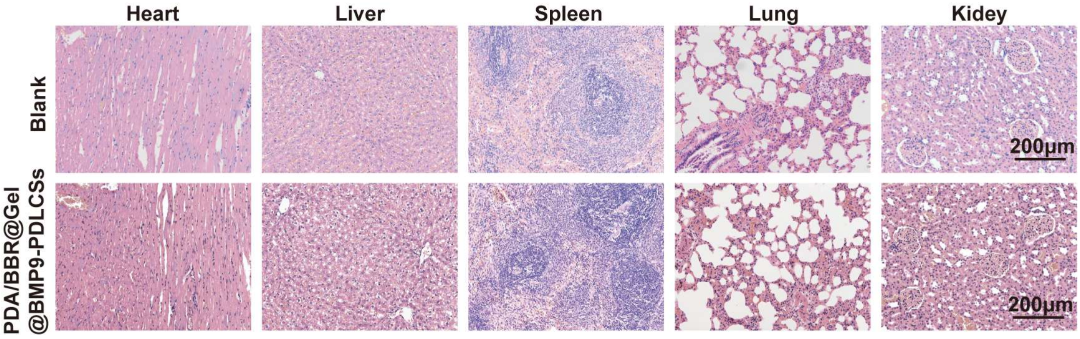

Figure S9. H&E staining of heart, liver, spleen, lung, and kidney in rats 4 weeks after implantation of PDA/BBR@Gel@BMP9-PDLCSs engineered microspheres.

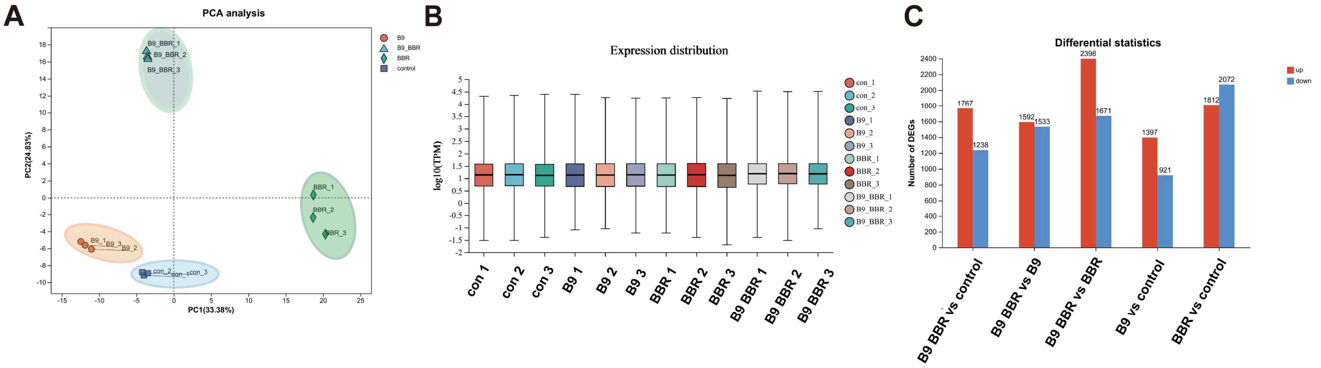

Figure S10. (A) PCA analysis of all samples. (B) Box plot of gene expression distribution of the dataset after normalization. (C) Histogram of significant difference statistics.

Table S1. List of qPCR primers of hPDLSCs

Gene Name	Direction	Sequence
**GAPDH**	Forward	GGAGTCCACTGGCGTCTTCA
	Reverse	GTCATGAGTCCTTCCACGATACC
**ALP**	Forward	CTTGACCTCCTCGGAAGACAC
	Reverse	CAGACCAAAGATAGAGTTGCCAC
**RUNX2**	Forward	GAACTGGGCCCTTTTTCAGA
	Reverse	CGGGGTGTAAGTAAAGGTGG
**OPN**	Forward	GCTTGGCTTATGGACTGAGG
	Reverse	GCTTGGCTTATGGACTGAGG
**IL-4**	Forward	TCTCACCTCCCAACTGCTTC
	Reverse	GTGTTCTTGGAGGCAGCAAA
**IL-6**	Forward	AAATTCGGTACATCCTCGACGG
	Reverse	GGAAGGTTCAGGTTGTTTTCTGC
**IL-10**	Forward	CCCTGTGAAAACAAGAGCAAGG
	Reverse	ACCCTGATGTCTCAGTTTCGT
**TNF-α**	Forward	GCCCATGTTGTAGCAAACCC
	Reverse	TATCTCTCAGCTCCACGCCA

Table S2 List of qPCR primers for mouse genes

Gene Name	Direction	Sequence
**Gapdh**	Forward	TGACCACAGTCCATGCCATC
	Reverse	GACGGACACATTGGGGGTAG
**IL-1β**	Forward	ATGCCACCTTTTGACAGTGATG
	Reverse	TGATGTGCTGCTGCGAGATT
**Tnf-α**	Forward	GGTGCCTATGTCTCAGCCTCTT
	Reverse	GCCATAGAACTGATGAGAGGGAG

**IL-6**	Forward	TGATGGATGCTACCAAACTGGA
	Reverse	TCTGTGACTCCAGCTTATCTCTTG
**IL-10**	Forward	GCAGCCTTGCAGAAAAGAGA
	Reverse	CTGGGAAGTGGGTGCAGTTA
**Tgf-β**	Forward	GACCTGGGTTGGAAGTGGAT
	Reverse	TTGGTTGTAGAGGGCAAGGA
**Ym-1**	Forward	AGAAGGGAGTTTCAAACCTGGT
	Reverse	CTCTTGCTGATGTGTGTAAGTGA
